# Synergistic Sunlight‐Activated Photodynamic and Near‐Infrared‐Induced Mild Photothermal Therapy for Infected Wound Healing Using Functionalized Nano‐Bi_2_WO_6_ Composites

**DOI:** 10.1002/advs.202522124

**Published:** 2026-04-10

**Authors:** Yihao Sun, Liangrui He, Zhijun Shen, Yuxin Zhang, Yizhang Tang, Xujiang Yu, Jisi Zheng, Moran Huang, Wanwan Li, Lei Wang

**Affiliations:** ^1^ Foot & Ankle Surgery Department of Orthopaedics Shanghai Sixth People's Hospital Affiliated to Shanghai Jiao Tong University School of Medicine Shanghai China; ^2^ Department of General Practice Shanghai Sixth People's Hospital Affiliated to Shanghai Jiao Tong University School of Medicine Shanghai China; ^3^ State Key Lab of Metal Matrix Composites School of Materials Science and Engineering Shanghai China; ^4^ Zhangjiang Institute for Advanced Study Shanghai Jiao Tong University Shanghai 200240 China; ^5^ Department of Oral Surgery Shanghai Ninth People's Hospital Shanghai Jiao Tong University School of Medicine Shanghai China

**Keywords:** antibacterial, immunomodulatory, macrophage polarization, photodynamic therapy (PDT), photothermal therapy (PTT)

## Abstract

Bacterial infection‐associated inflammation and drug resistance severely impede effective wound healing. Herein, we report a multifunctional Bi_2_WO_6_:Yb,Er@CuS@CS nanoplatform that integrates photodynamic therapy (PDT), near‐infrared (NIR)‐triggered mild photothermal therapy (PTT), and Cu^2^^+^ release for infected wound repair. Yb^3+^/Er^3+^ codoping endows Bi_2_WO_6_ with broadened visible–NIR light absorption and enhanced charge separation, enabling efficient reactive oxygen species generation under simulated sunlight. Surface‐decorated CuS nanoparticles provide robust photothermal conversion under NIR irradiation, while chitosan modification improves biocompatibility and antibacterial performance. The nanoplatform exhibits > 99% antibacterial efficacy against both Methicillin‐resistant *Staphylococcus aureus* (MRSA) and *Escherichia coli* and effectively disrupts bacterial biofilms under combined light activation. Importantly, precisely controlled NIR‐induced mild thermal therapy (≈42°C) reprograms macrophage polarization from a proinflammatory M1 phenotype toward a reparative M2 phenotype. Transcriptomic analysis reveals that this immunomodulatory effect is associated with the downregulation of PI3K–Akt, TNF, and NF–κB signaling pathways. In vivo studies using MRSA‐infected wound models demonstrate accelerated wound healing, enhanced angiogenesis, collagen deposition, and minimal systemic toxicity. This work presents a sunlight/NIR‐activated therapeutic strategy that bridges antibacterial treatment and immune‐regulated tissue regeneration for infected wound healing.

## Graphical abstract

This work proposes a multifunctional Bi_2_WO_6_:Yb,Er@CuS@CS nanocomposite that integrates photodynamic therapy (PDT), near‐infrared‐triggered photothermal therapy (PTT), and Cu^2+^ release for infected wound repair. The nanocomposite demonstrates enhanced antibacterial efficacy via combined PDT, PTT, and Cu^2+^ release, effective immunomodulatory by mild thermal therapy and Cu^2+^ release, and significantly accelerated infected wound healing in vivo.

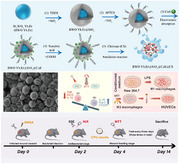

## 1 Introduction

The widespread use and increasing misuse of antibiotics have led to the global emergence of antibiotic‐resistant bacteria [1, 2, 3]. Moreover, wound healing complicated by bacterial infection also yields suboptimal therapeutic outcomes due to the absence of a suitable skin repair microenvironment [4]. Generally, when skin integrity is compromised, bacteria (including antibiotic‐resistant strains) will colonize the wound surface, forming biofilm barriers that trigger chronic infection [5, 6]. This results in delayed or nonhealing wounds, exacerbating patient suffering and economic burdens. Additionally, during the initial phase of the disease, the immune system is activated, creating a localized inflammatory storm to eliminate the bacteria [7, 8]. However, regardless of bacterial clearance, immune cells release excessive inflammatory mediators, perpetuating the inflammatory storm and impeding tissue regeneration. Thus, developing a multifunctional strategy to treat chronic infections caused by multidrug‐resistant bacteria and promote wound healing is highly necessary.

Recently, photodynamic therapy (PDT) and photothermal therapy (PTT) have emerged as promising alternatives due to their low risk of inducing resistance and operational convenience, with advancements in nanomaterials and photosensitizers [9, 10]. Classic photosensitizers are inorganic nanomaterials (e.g., TiO_2_, ZnO, Cu_2_O), which under ultraviolet/blue light irradiation produce free electrons and holes that react with oxygen or water to generate reactive oxygen species (ROS) (e.g.,·OH, ·O_2_^−^), inhibiting bacterial growth and promoting wound healing [11, 12, 13]. However, unmodified nanomaterials exhibit relatively low PDT efficiency. Bi_2_WO_6_ has been utilized as a photosensitizer due to its narrower bandgap (∼2.7 eV), stronger visible‐light response, superior chemical stability, enhanced biocompatibility, and lower cost compared to conventional alternatives [14, 15, 16]. Upon excitation by light with energy exceeding its bandgap, Bi_2_WO_6_ generates electron‐hole pairs (e^−^–h^+^), which react with surface‐adsorbed O_2_ and H_2_O to produce ROS (·OH and ·O_2_^−^), enabling PDT. Recent efforts to enhance its photocatalytic activity include doping with rare‐earth ions (Yb^3+^, Er^3+^), introducing F^−^, or constructing Z‐scheme heterojunctions [17, 18, 19]. However, studies on its in vivo antibacterial performance remain limited.

PDT and PTT synergistic therapy can enhance antibacterial efficacy while mitigating the adverse effects of ROS overproduction on wound healing [20, 21]. PTT utilizes photothermal agents to induce localized hyperthermia under near‐infrared (NIR) irradiation, thereby effectively eliminating bacteria while circumventing concerns related to antibiotic resistance and biocompatibility, making it a current research focus [22]. Based on the achieved temperature, PTT can be classified into mild hyperthermia (< 42°C), thermotherapy (42–48°C), and thermal ablation (> 48°C). Copper sulfide (CuS), a narrow‐bandgap semiconductor chalcogenide, exhibits both photocatalytic and superior photothermal properties [23]. Unlike conventional PTT, which relies solely on NIR irradiation, CuS‐based strategies enhance antibacterial efficacy under accessible conditions while minimizing thermal injury [24]. Moreover, CuS nanoparticles possess an intrinsic pathogen‐eradication potential. The released Cu^2+^, an essential human trace element, further promotes tissue repair by stimulating healing pathways [25]. Notably, PTT serves not only as an antibacterial intervention, but mounting evidence from recent years has also demonstrated that mild hyperthermia (< 42°C) plays a pivotal role in modulating immune cells and promoting tissue regeneration [23, 26]. For instance, in bone injury repair, cyclical mild thermotherapy treatment has been reported to modulate the local inflammatory microenvironment via the PI3K–Akt1 signaling pathway, enhancing osteogenic differentiation of mesenchymal stem cells [27]. However, research on the anti‐inflammatory properties of “mild thermal therapy” in infected skin wounds remains limited.

Inflammatory cells, particularly macrophages, play a critical role during wound healing in infected wounds [28, 29]. Proinflammatory (M1) macrophages exacerbate tissue damage by releasing excessive cytokines (e.g., TNF‐α, IL‐6), whereas anti‐inflammatory (M2) macrophages facilitate inflammation resolution, angiogenesis, and collagen remodeling [30, 31]. Additionally, Cu^2+^ has been reported to modulate macrophage polarization. The combination of mild hyperthermia with Cu^2+^ is expected to amplify the anti‐inflammatory regulation synergistically [32]. Therefore, by adjusting the NIR irradiation intensity (the “Knob effect”) to achieve precise thermal control, a bridge is established between early antibacterial effects and later tissue repair, providing a promising example for managing infected wounds. Chitosan (CS) is a nontoxic, biosafe, and biodegradable natural cationic polymer. It adsorbs onto negatively charged bacterial biofilms via electrostatic interactions, thereby disrupting bacterial motility and biofilm integrity [33, 34]. To further enhance antibacterial performance and biocompatibility, the nanoplatform was modified with an outer chitosan coating.

**SCHEME 1:**
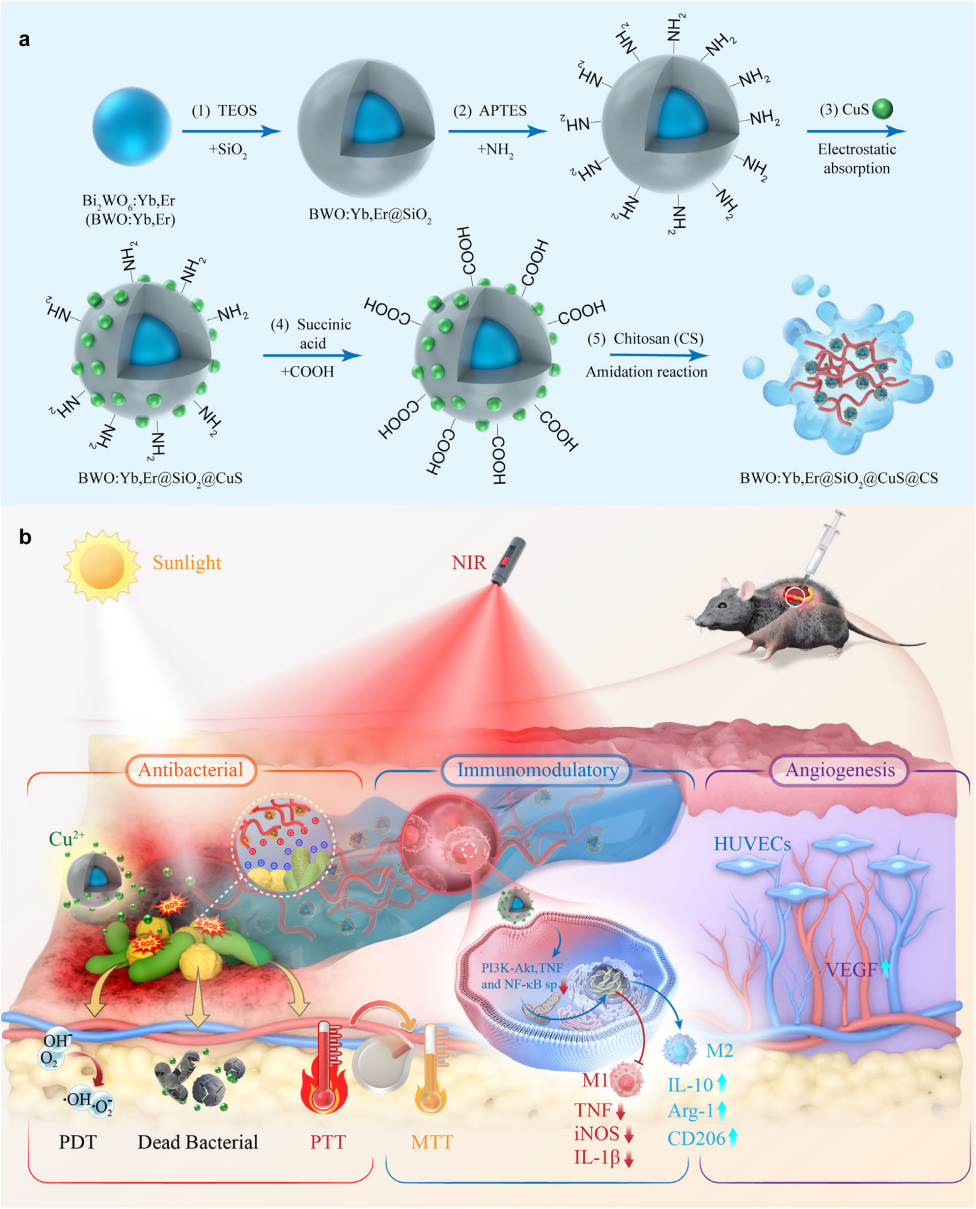
A multifunctional intelligent nanoplatform serves as a novel strategy for repairing infected skin wounds. (a) Schematic illustration of the fabrication of Bi_2_WO_6_:Yb,Er@CuS@CS intelligent nanoplatform: (1) hydrothermal synthesis of Bi_2_WO_6_:Yb,Er nanoparticles followed by SiO_2_ coating via TEOS hydrolysis; (2) surface amination through APTES hydrolysis; (3) electrostatic assembly between positively charged BWO:Yb,Er@SiO_2_–NH_2_ and negatively charged citrate‐modified CuS nanoparticles; (4) conversion of excess amino groups into carboxyl groups using succinic anhydride; and (5) chitosan conjugation onto BWO:Yb,Er@SiO_2_@CuS via an amidation reaction. (b) Its application for infected skin wounds therapy. (TEOS: tetraethylorthosilicate, APTES: (3‐Aminopropyl) triethoxysilane, NIR: near‐infrared laser, PDT: photodynamic therapy, PTT: photothermal therapy, MTT: mild thermal therapy, PI3K–Akt, TNF, and NF–κB sp: PI3K–Akt, TNF, and NF–κB signaling pathways, M1: M1 macrophage and M2: M2 macrophage).

Herein, we developed a combined PTT/PDT/Cu^2+^ approach using Bi_2_WO_6_:Yb,Er@CuS@CS nanoplatforms, aiming to achieve “mild therapy” to targeted antibacterial and wound healing (Scheme 1). By simulating sunlight to excite Bi_2_WO_6_:Yb,Er and generate reactive oxygen species, combined with the photothermal effect induced by near‐infrared radiation, and incorporating Cu^2+^ to achieve triple antibacterial action. Subsequently, the “Knob effect” was utilized to attain mild thermotherapy treatment (MTT), thereby reprogramming the immune microenvironment and promoting vascular regeneration. Furthermore, RNA sequencing analysis elucidated that the NIR‐induced MTT promoted the polarization of macrophages toward the M2 phenotype by downregulating the PI3K–Akt, TNF, and NF–κB signaling pathways, thereby revealing its immunomodulatory mechanism. This finding may provide a novel therapeutic strategy for infected skin wounds.

## 2 Results and Discussion

### 2.1 Preparation and Characterization of Bi_2_WO_6_:Yb,Er@CuS@CS

We used Bi_2_WO_6_ nanoparticles with high photocatalytic activity as a matrix and doped them with ytterbium and erbium ions to enhance the absorption intensity and range of white light [35, 36]. Then, we coated the Bi_2_WO_6_:Yb,Er (BWO:Yb,Er) nanoparticles with SiO_2_ to impart the amino group and biocompatibility. Negatively charged CuS nanoparticles decorated the surface of positively charged BWO:Yb,Er nanoparticles by electrostatic interaction [37]. The nanostructures were further modified with CS to enhance biocompatibility and antibacterial ability.

**FIGURE 1:**
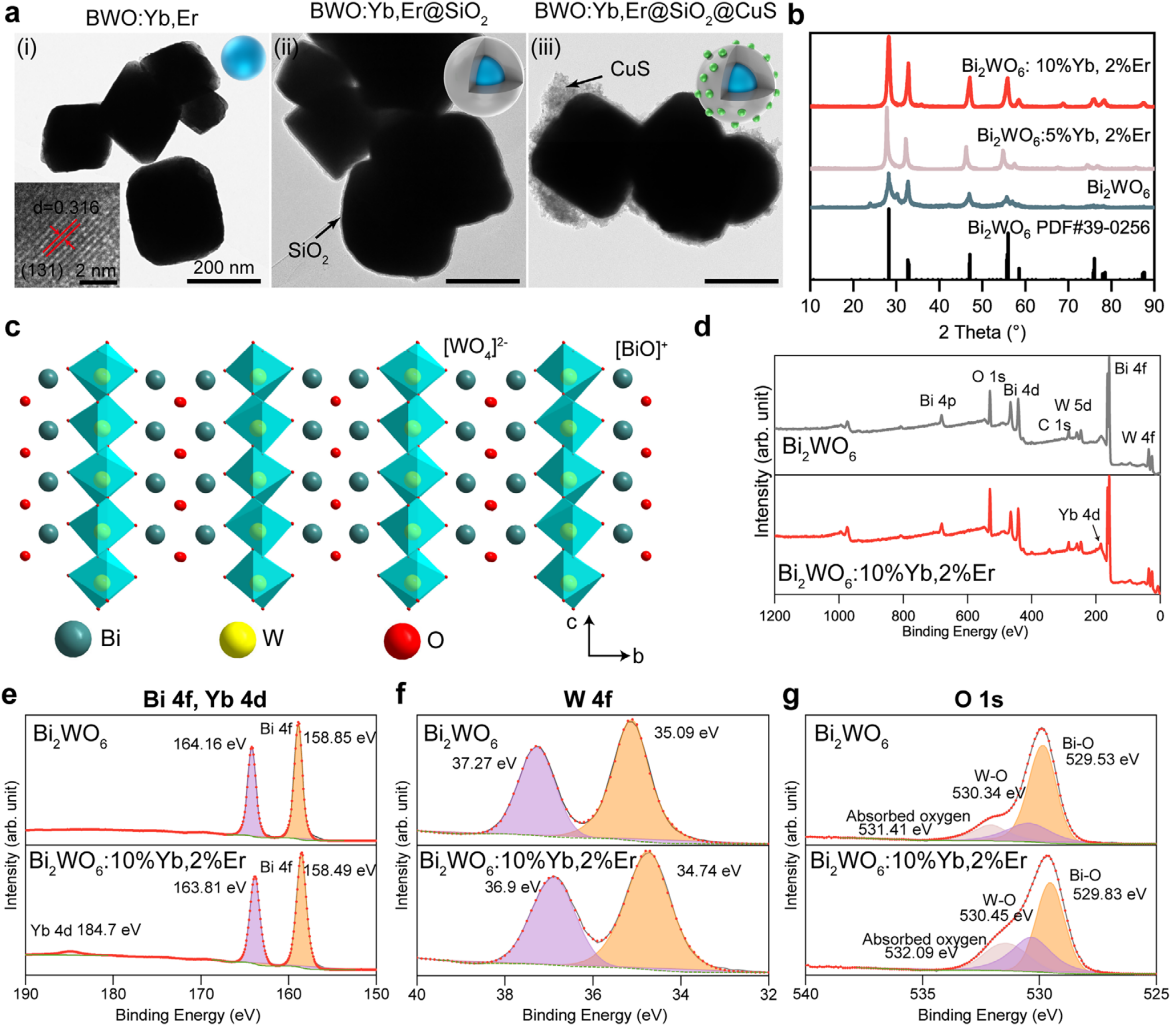
(a) Transmission electron microscope (TEM) images and (b) X‐ray diffraction (XRD) patterns of Bi_2_WO_6_:10%Yb,2%Er, Bi_2_WO_6_:10%Yb,2%Er@SiO_2_, and Bi_2_WO_6_:10%Yb,2%Er@SiO_2_@CuS nanoplatforms (inset image in (i): High‐resolution TEM (HRTEM) of BWO:Yb,Er). (c) Crystal structure of Bi_2_WO_6_. XPS spectra of pure Bi_2_WO_6_ and Bi_2_WO_6_:10%Yb,2%Er, (d) survey scan, (e) Bi 4f, (f) W 4f, and (g) O 1s.

Bi_2_WO_6_ nanoparticles with varying Yb^3+^ and Er^3+^ doping concentrations were synthesized using the hydrothermal method [38]. Transmission electron microscope (TEM) images showed that the pure Bi_2_WO_6_ had nanosheet structures with a size of ∼50 nm [39], whereas doping with Yb^3+^ and Er^3+^ showed nanoparticles with a size of ∼120 nm (Figure 1b). TEM images show that the CuS satellites can be firmly attached to the surface of the BWO:Yb,Er@SiO_2_–NH_2_ core due to the beneficial electrostatic adsorption, resulting in the construction of the core–satellite structure (Figure 1a). Additionally, X‐ray diffraction (XRD) patterns showed that the synthesized materials corresponded to the diffraction peaks of the orthorhombic phase Bi_2_WO_6_ (PDF 39‐0256) (Figure 1b). Meanwhile, high‐resolution transmission electron microscopy (HRTEM) of Bi_2_WO_6_:Yb,Er showed a lattice stripe spacing of 0.316 nm, corresponding to the (131) crystal plane. The XRD patterns also revealed the crystallization performance of the three synthesized materials. After doping with Yb^3+^ and Er^3+^, the diffraction peaks of Bi_2_WO_6_:Yb,Er nanoparticles were sharper than those of Bi_2_WO_6_ nanosheets, suggesting that the crystallization behavior of Bi_2_WO_6_:Yb,Er nanoparticles was better than that of Bi_2_WO_6_ nanosheets, which was consistent with the TEM results. High‐angle annular dark‐field imaging, energy‐dispersive X‐ray spectroscopy (EDS) mapping, and inductively coupled plasma (ICP) analysis also confirmed these findings (Figure S1 and Table S1). The crystal structure of Bi_2_WO_6_ monolayers forming a sandwich‐like structure [BiO]^+^–[WO_4_]^2−^–[BiO]^+^ exhibited excellent photodynamic properties and was an ideal system for isolating subsurface defects (Figure 1c). Moreover, this doping behavior of Yb^3+^ and Er^3+^ ions constructed more electron traps, which prolonged the lifetime of photogenerated charge carriers (electrons and holes) and improved their separation efficiency [40]. Meanwhile, Yb^3+^ and Er^3+^ ions tuned the band structure of the material, leading to a change in the bandgap energy and an increase in the absorption of visible and NIR light, resulting in a significant improvement in the overall photodynamic properties [41]. X‐ray photoelectron spectroscopy (XPS) analysis revealed the surface chemical microenvironment of the as‐prepared Bi_2_WO_6_ and Bi_2_WO_6_:10%Yb,2%Er (Figure 1d–g). The survey XPS spectra of pure Bi_2_WO_6_ and Bi_2_WO_6_:10%Yb,2%Er, and the primary presence of Bi, W, O, and Yb (Figure 1d). However, the element of Er was not detected because of the low doping concentration. In the high‐resolution XPS spectrum of Bi 4f in pure Bi_2_WO_6_, the peaks at 158.85 and 164.16 eV correspond to the ^4^f_5/2_ and ^4^f_7/2_ orbitals of Bi^3+^, respectively (Figure 1e). The peaks of Bi ^4^f_5/2_ and ^4^f_7/2_ in Bi_2_WO_6_:10%Yb,2%Er were shifted toward lower energies by about 0.35 eV. This shift could be attributed to the introduction of Yb or Er in the Bi_2_WO_6_ lattice, and the increased electron density around Bi^3+^ after doping with Yb^3+^ and Er^3+^ ions [42]. Also, the characteristic peaks of W ^4^f_7/2_ and ^4^f_5/2_ were observed at 35.09 and 37.27 eV, respectively, which can be ascribed to W^6+^ (Figure 1f). The binding energies of W ^4^f_7/2_ and ^4^f_5/2_ of Bi_2_WO_6_:10%Yb,2%Er are slightly lower than those of pure Bi_2_WO_6_ [43]. The asymmetric O 1s peak of pure Bi_2_WO_6_ could be divided into three peaks at 529.53, 530.34, and 531.41 eV, ascribing to the coordination of oxygen (Bi–O, W–O, and surface adsorbed oxygen, respectively) (Figure 1g). However, the binding energy of O 1s in Bi_2_WO_6_:10%Yb,2%Er is shifted to higher energies by 0.3 eV compared to pure Bi_2_WO_6_, probably due to the formation of Bi–O–Yb and Bi–O–Er bonds. These results indicated that the formation of oxygen vacancies in the [BiO]^+^ layer after doping with Yb^3+^ and Er^3+^ improves carrier separation efficiency, enlarges the visible light absorption range, and facilitates reactant activation [44].

**FIGURE 2:**
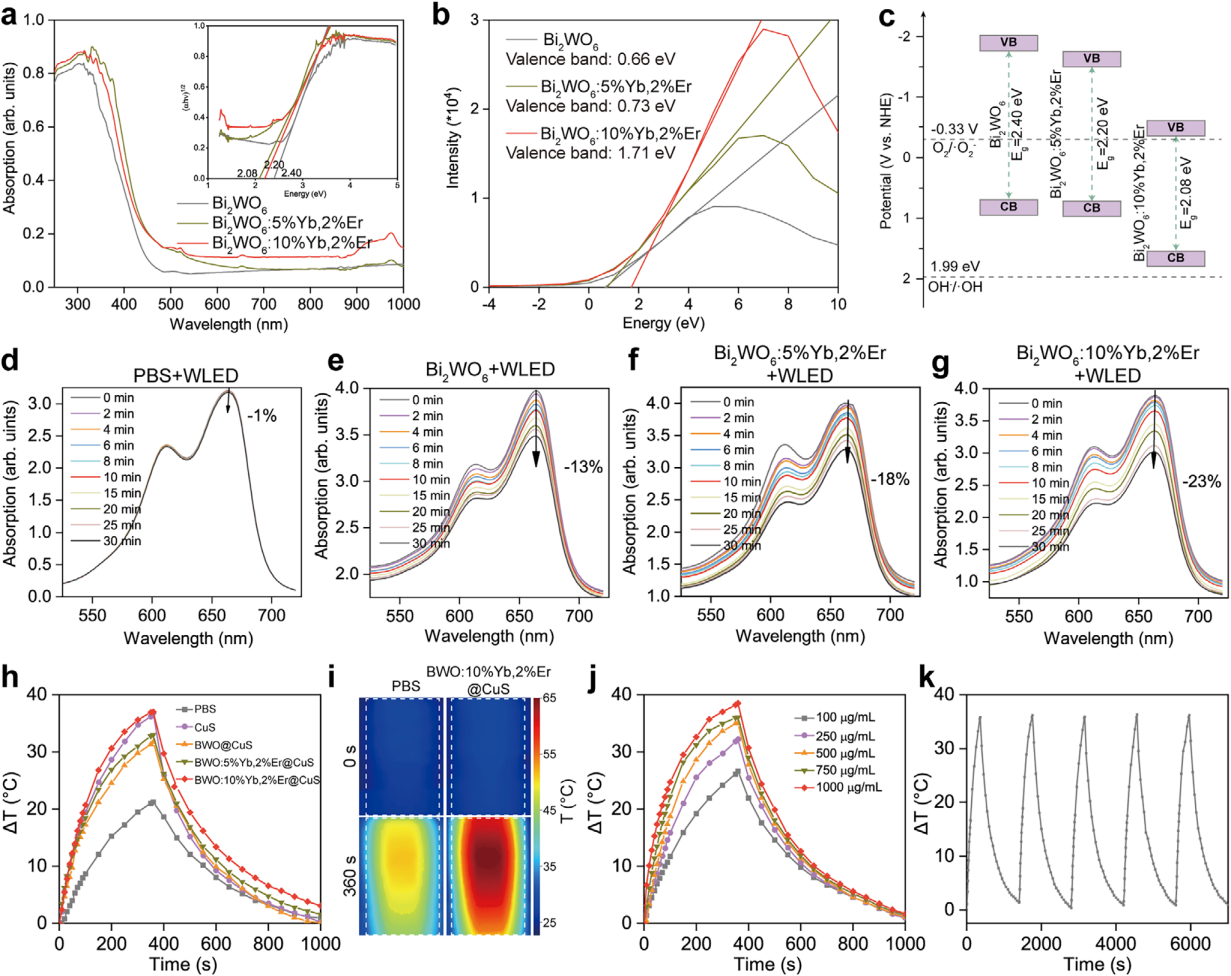
(a) UV–vis absorption curves of the pure Bi_2_WO_6_, Bi_2_WO_6_:5%Yb,2%Er, and Bi_2_WO_6_:10%Yb,2%Er nanoparticles. The inset showed the bandgap energy (*E*_g_) determination from the Tauc plot. (b) Valence band XPS spectra of the pure Bi_2_WO_6_, Bi_2_WO_6_:5%Yb,2%Er, and Bi_2_WO_6_:10%Yb,2%Er nanoparticles. (c) Proposed band structures of the pure Bi_2_WO_6_, Bi_2_WO_6_:5%Yb,2%Er, and Bi_2_WO_6_:10%Yb,2%Er nanoparticles. UV–vis absorption spectra of methylene blue (MB) degradation using (d) PBS, (e) pure Bi_2_WO_6_, (f) Bi_2_WO_6_:5%Yb,2%Er, and (g) Bi_2_WO_6_:10%Yb,2%Er. (h) Photothermal heating curves recording the temperature variations of PBS, CuS, Bi_2_WO_6_@CuS, Bi_2_WO_6_:5%Yb,2%Er@CuS, and Bi_2_WO_6_:10%Yb,2%Er@CuS under 980 nm laser irradiation (1 W/cm^2^). (i) Infrared thermographic maps of PBS and Bi_2_WO_6_:10%Yb,2%Er@CuS in tubes after irradiation for 0 and 6 min under the 980 nm laser (1 W/cm^2^). (j) Temperature curves of different concentrations of Bi_2_WO_6_:10%Yb,2%Er nanoparticles in aqueous solution upon laser irradiation at 980 nm (1 W/cm^2^). (k) Temperature profile of Bi_2_WO_6_:10%Yb,2%Er nanoparticles upon cycled laser irradiation at 980 nm (1 W/cm^2^).

To confirm the significant role of codoped lanthanide ions during photodynamic reactions, the absorption properties of pure Bi_2_WO_6_, Bi_2_WO_6_:5%Yb,2%Er, and Bi_2_WO_6_:10%Yb,2%Er were investigated (Figure 2a). The absorption edge of the as‐prepared materials was red‐shifted upon doping with lanthanide ions. Notably, Bi_2_WO_6_:10%Yb,2%Er exhibited the visible–NIR adsorption range, in contrast to the other materials. Moreover, the peak at ∼975 nm in the absorption curves can be attributed to the absorption of Yb^3+^ [45, 46], which also confirms the success of doping. The bandgap energy (*E*_g_) values can be estimated using the equation [47]:

$$\alpha h\upsilon =B{(h\upsilon -Eg)}^{n/2}$$

where *α*, *h*, *B*, *E*_g_, and *n* represent the absorption coefficient, the Planck constant, the constant of proportionality, the bandgap, and an integer, respectively. The coefficient *n* is determined by the optical transition type of the semiconductor photocatalyst (*n* = 1 or 4), and in the case of the Bi_2_WO_6_ system, *n* = 4, because the Bi_2_WO_6_ is an indirect semiconductor [36, 48]. Thus, the bandgaps (*E*_g_) of pure Bi_2_WO_6_, Bi_2_WO_6_:5%Yb,2%Er, and Bi_2_WO_6_:10%Yb,2%Er were determined to be 2.40, 2.20, and 2.08 eV, respectively (Figure 2a). The bandgaps of Yb^3+^, Er^3+^ codoped Bi_2_WO_6_ were relatively narrower than that of pure Bi_2_WO_6_, which was of benefit to their white‐light‐driven photodynamic performance. Meanwhile, we utilized the XPS VB spectra to determine the valence band (VB) potential of pure Bi_2_WO_6_, Bi_2_WO_6_:5%Yb,2%Er, and Bi_2_WO_6_:10%Yb,2%Er [49]. In general, a valence band position close to the oxidation potential of OH^−^/·OH facilitates the direct oxidation of surface hydroxyl groups by valence band holes (h^+^), while a conduction band position near the reduction potential of O_2_/O_2_^−^ is conducive to the reduction of adsorbed O_2_ by conduction band electrons (e^−^) [50, 51, 52]. The VB edge potentials of pure Bi_2_WO_6_, Bi_2_WO_6_:5%Yb,2%Er, and Bi_2_WO_6_:10%Yb,2%Er were 0.66, 0.73, and 1.71 eV (Figure 2b). Correspondingly, the minimum values of the conduction band (CB) can be calculated to be −1.74, −1.47, and −0.37 eV, respectively. The valence bands of pure Bi_2_WO_6_, Bi_2_WO_6_:5%Yb,2%Er, and Bi_2_WO_6_:10%Yb,2%Er were more negative than E(O_2_/·O_2_^−^) (−0.33 eV), so the photogenerated electron (e^−^) transfers quickly to the photocatalyst surface and reacts with O_2_ to generate ·O_2_^−^ (Figure 2c) [53]. Simultaneously, the valence bands of pure Bi_2_WO_6_, Bi_2_WO_6_:5%Yb,2%Er, and Bi_2_WO_6_:10%Yb,2%Er were more negative than E(OH^−^/·OH) (1.99 eV), which was unfavorable for the oxidation of OH^−^ radicals to ·OH. Notably, the valence band of Bi_2_WO_6_:10%Yb,2%Er was closer to E(OH^−^/·OH) than those of pure Bi_2_WO_6_ and Bi_2_WO_6_:5%Yb,2%Er, thus facilitating the formation of ·OH. By optimizing the doping concentrations of Yb^3+^ and Er^3+^, the bandgap the doped Bi_2_WO_6_ was reduced through the introduction of impurity energy levels, thereby broadening the light response range under white‐light irradiation. Meanwhile, the positions of the conduction band minimum and valence band maximum were favorably modulated to match the redox potentials of O_2_/O_2_^−^ and OH^−^/·OH, respectively, which enhanced the generation of ROS and consequently improved the PDT efficiency. Furthermore, the photocatalytic performance of the as‐prepared nanoparticles was analyzed using methylene blue (MB) photodegradation under white‐light excitation. A blank experiment was first performed to clarify MB's self‐degradation behavior. The absorption intensity at the peak of 660 nm did not decrease significantly (1%) when the MB solution was irradiated with white light for 30 min (Figure 2d). The absorption intensity of the MB solution at 660 nm decreased by 13%, 18%, and 23% when pure Bi_2_WO_6_, Bi_2_WO_6_:5%Yb,2%Er, and Bi_2_WO_6_:10%Yb,2%Er were used as photocatalysts, respectively (Figure 2e–g). The photodegradation rate of MB showed an increasing trend with the prolongation of irradiation time, indicating that the photocatalytic activity of Bi_2_WO_6_ nanoparticles could be enhanced by Er^3+^ and Yb^3+^ codoping. In particular, Bi_2_WO_6_:10%Yb,2%Er nanoparticles exhibited excellent reactive oxygen species yield under white light irradiation. After conjugating with a photothermal agent of CuS nanoparticles, we further investigated the photothermal conversion efficiency (PTCE) of Bi_2_WO_6_@CuS, Bi_2_WO_6_:5%Yb,2%Er@CuS, and Bi_2_WO_6_:10%Yb,2%Er@CuS nanoparticles. After 6 min of irradiation of a 980 nm NIR laser (1 W/cm^2^), the temperature of PBS increased by 21.2°C, which was attributed to the absorption of 980 nm light by water. Meanwhile, the temperature of CuS, Bi_2_WO_6_@CuS, Bi_2_WO_6_:5%Yb,2%Er@CuS, and Bi_2_WO_6_:10%Yb,2%Er@CuS increased by 36.8, 31.7, 33, and 37°C, respectively (Figure 2h), resulting in the corresponding temperature increments per minute for them being 6.1, 5.3, 5.5, and 6.2°C/min_,_ which were sufficient for the photothermal killing of pathogenic microorganisms [54]. The temperature increment per minute of Bi_2_WO_6_:10%Yb,2%Er@CuS was higher than that of other nanoplatforms, which could be attributed to the conversion of 980 nm light absorption by Yb^3+^ ions into phonon vibrations [55, 56]. The infrared thermal images of PBS and Bi_2_WO_6_:10%Yb,2%Er@CuS nanoparticles also confirmed the result (Figure 2i). The PTCEs were approximately 48.5%, 36.4%, 27.8%, and 47.1% for CuS, Bi_2_WO_6_@CuS, Bi_2_WO_6_:5%Yb,2%Er@CuS, and Bi_2_WO_6_:10%Yb,2%Er@CuS, respectively. In addition, the temperature of the as‐prepared Bi_2_WO_6_:10%Yb,2%Er@CuS nanoplatforms increased with increasing irradiation time in a concentration‐dependent manner (Figure 2j). The Bi_2_WO_6_:10%Yb,2%Er@CuS nanoplatform exhibited excellent photothermal stability, as its photothermal conversion capacity remained unchanged after several heating–cooling cycles under laser irradiation at a wavelength of 980 nm, which also indicated that the temperature increase would not detach the CuS from the surface of Bi_2_WO_6_:10%Yb,2%Er nanoparticles (Figure 2k). To evaluate the stability of the nanoplatform and the potential risk of metal leaching during use, the nanoplatforms were incubated in MES buffer, which maintained good stability during 5 days (Figure S2) Moreover, metal ion release analysis revealed negligible leaching over the tested period, which can be attributed to the stable crystal lattice and the surface protection provided by the coating layer (Table S2). These results demonstrate that the nanoplatform exhibits satisfactory stability and a low risk of metal leaching for biomedical applications.

### 2.2 In Vitro Antibacterial Activity of Bi_2_WO_6_:Yb,Er@CuS@CS

We evaluated the cytotoxicity of Bi_2_WO_6_:Yb,Er@CuS@CS on human umbilical vein endothelial cells (HUVECs) using CCK‐8 and live/dead staining methods. Results showed no significant toxicity at 200 µg/mL after 24, 48, and 72 h, with or without stimulation (30 min simulated sunlight exposure (SSE)+15 min NIR) (Figure S3a–c). Instead, a slight proliferative effect was observed, possibly due to the release of Cu^2+^. Live/dead staining confirmed high cell viability at concentrations of ≤ 200 µg/mL, with increased proliferation observed after 48 h. Furthermore, TRITC‐phalloidin staining revealed normal cytoskeleton morphology at 200 µg/mL postintervention (Figure S3d). Overall, Bi_2_WO_6_:Yb,Er@CuS@CS demonstrates favorable biocompatibility.

**FIGURE 3:**
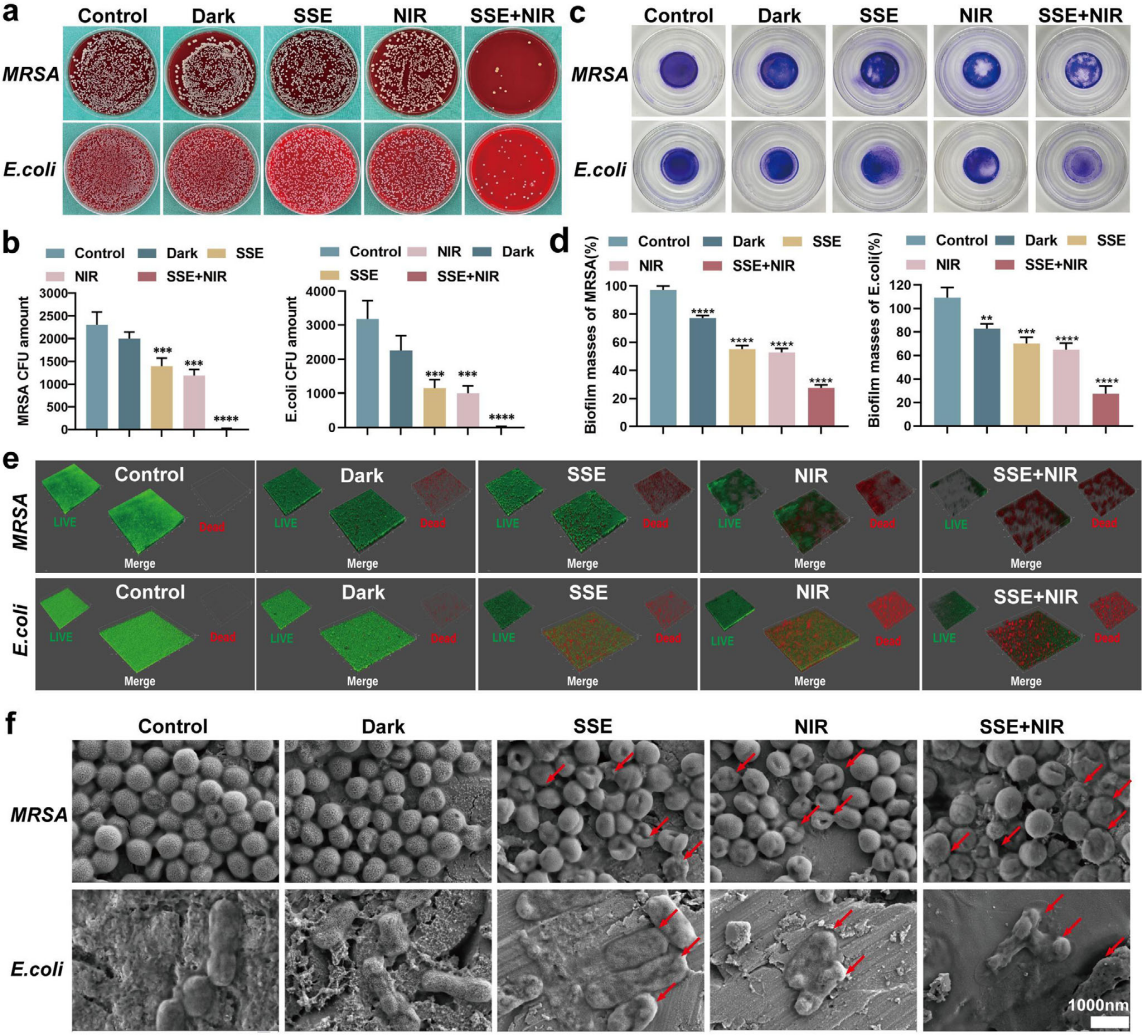
The Bi_2_WO_6_:Yb,Er@CuS@CS nanoplatform exhibits in vitro antibacterial performance against MRSA and *E. coli*. (a) Photographs of bacterial colonies grown on sheep blood agar plates from different treatment groups, with (c) quantitative analysis of CFU counts, *n* = 3. (b) Crystal violet staining images of biofilms from different treatment groups, with (d) absorbance measurements of stained biofilms at 550 nm, *n* = 3. (e) Confocal microscopy images of MRSA and *E. coli* subjected to different treatments: PI (red): dead bacteria, SYTO9 (green): live bacteria. (f) SEM images showing bacterial morphology of MRSA and *E. coli* after different treatments. Red arrows: shriveled/lysed bacteria. (ns, *p* > 0.05; *, *p* < 0.05; **, *p* < 0.01; ***, *p* < 0.001; ****, *p* < 0.0001).

To assess the in vitro antibacterial activity of Bi_2_WO_6_:Yb,Er@CuS@CS under various intervention conditions, Methicillin‐resistant *Staphylococcus aureus* (MRSA, a Gram‐positive bacterium) and *Escherichia coli* (*E. coli*, a Gram‐negative bacterium) were utilized. First, we verified the electrostatic adsorption effect of the CS coating. As shown in Figure S4a, after standing for 5 min, the mixture containing Bi_2_WO_6_:Yb,Er@CuS@CS exhibited slight precipitation, and the supernatant appeared clearer compared to that of the Bi_2_WO_6_:Yb,Er@CuS group. This phenomenon became more pronounced after 10 min of standing. Furthermore, scanning electron microscopy (SEM) images (Figure S4b) revealed that in biofilms treated with Bi_2_WO_6_:Yb,Er@CuS@CS (200 µg/mL), a greater amount of the material adhered to the biofilm surface compared to those treated with Bi_2_WO_6_:Yb,Er@CuS (200 µg/mL). Then, the dilution method was employed to quantify the colony‐forming units on the coated plates and to determine the antibacterial efficacy. As shown in Figure 3a,b, in a completely dark environment (Dark), the antibacterial efficacy of Bi_2_WO_6_:Yb,Er@CuS@CS is notably limited, with efficacy rates of 12.9% against MRSA and 29.9% against *E. coli*, respectively. Following the application of SSE or NIR radiation independently, the antibacterial efficiency was increased. The SSE group demonstrated antibacterial efficiency rates of 39.3% against MRSA and 63.5% against *E. coli*, while the NIR group exhibited rates of 48.3% and 68.4%, respectively. Following the combined intervention of SSE and NIR, the antibacterial efficiency was significantly enhanced. The results indicate that this method can inhibit the growth of 99.1% of MRSA and 99.4% of *E. coli*. These results suggest that the Bi_2_WO_6_:Yb,Er@CuS@CS nanoplatform exhibits robust antibacterial activity.

However, biofilms are primarily composed of an extracellular matrix secreted by the bacteria themselves, which inhibits the penetration of antibiotics and subsequently induces the rapid development of bacterial resistance. Consequently, the ability to inhibit and disrupt biofilms serves as a critical criterion for assessing the in vitro antibacterial activity of the nanoplatforms. To evaluate the antibiofilm efficacy of the nanoplatform, MRSA and *E. coli* were selected as representative strains for infectious skin wounds, and monospecies biofilms were cultivated in vitro. The ability to inhibit and disrupt biofilms was assessed using the crystal violet staining method, with the OD550 value employed to quantify biofilm biomass. As shown in Figure 3c, among all groups, those subjected to treatment with SSE+NIR exhibited the lowest OD550 values for both MRSA and *E. coli*, at 28.5% ± 2.2% and 24.9% ± 3.5%, respectively (Figure 3d), indicating optimal antibiofilm efficacy.

In order to distinguish the contributions of ROS from sunlight (PDT), heat from NIR light, and Cu^2+^ release to the antibacterial efficacy, we separately tested the effects of CuS@CS+Dark, Bi_2_WO_6_:Yb,Er@CS+SSE, and CuS@CS+NIR in antibacterial experiments. By comparing the colony counts and antibiofilm crystal violet staining results of the CuS@CS+Dark, Bi_2_WO_6_:Yb,Er@CS+SSE, and CuS@CS+NIR groups (Figure S5), it can be concluded that the antibacterial effect of Cu^2+^ release is relatively limited, though it still shows a statistically significant difference compared to the control group. In contrast, the antibacterial effects of PDT alone and the combined effect of Cu^2+^ and PTT are more substantial. Compared to the control group, the staining rates of MRSA biofilm were reduced to 67.13% ± 3.11% and 49.22% ± 1.94%, respectively, while those of *E. coli* biofilm were reduced to 65.29% ± 3.16% and 54.54% ± 2.81%, respectively.

Meanwhile, we performed fluorescent imaging of the ROS generated by PDT and further distinguished the types of ROS produced. As shown in Figure S6a,b, we employed the fluorescent probe DCFH‐DA to capture ROS fluorescence images and perform quantitative analysis of the control group, the SSE group, the Bi_2_WO_6_:Yb,Er@CuS@CS+Dark group, and the Bi_2_WO_6_:Yb,Er@CuS@CS+SSE group. The results indicate that simulated sunlight irradiation alone on Raw 264.7 macrophages did not generate detectable ROS. After adding Bi_2_WO_6_:Yb,Er@CuS@CS, a small amount of ROS was produced, which showed a significant difference compared to the control group. This effect is likely attributable to the intrinsic stimulatory effect of the material on cells. Finally, upon exposing the material to simulated sunlight, a substantial amount of ROS was generated, and the increase was statistically significant compared to the group with only the nanocomposite added. These ROS contributed to the PDT effect observed in the antibacterial experiments. Furthermore, we differentiated the types of ROS generated using three fluorescent probes (Figure S6c): DHE, HPF, and SOSG, which specifically detect superoxide anion (·O_2_^−^), hydroxyl radicals (·OH), and singlet oxygen (^1^O_2_), respectively. The results demonstrated that the ROS generated by Bi_2_WO_6_:Yb,Er@CuS@CS under simulated sunlight exposure mainly consisted of superoxide anions, along with a small amount of hydroxyl radicals. The level of singlet oxygen produced was relatively low and did not show a statistically significant difference.

Furthermore, to obtain a clearer understanding of the role of the Bi_2_WO_6_:Yb,Er@CuS@CS nanoplatform in disrupting biofilm accumulation, LIVE/DEAD staining was conducted on biofilms following various interventions. Live bacteria were stained green using SYTO 9, while dead bacteria were stained red with propidium iodide (PI). Subsequently, confocal microscopy was employed for imaging. Figure 3e shows the antibiofilm effects of different treatments on two types of monospecies biofilms. The Control group exhibited nearly complete live bacterial biofilms, with intense green staining, and almost no red‐stained dead bacteria were observed. In the Dark group, the biofilm formed by live bacteria remained intact; however, a small number of bacteria, which had been stained red, were observed. In contrast, the live bacterial biofilms in the SSE and NIR groups exhibited substantial damage compared to the control group, accompanied by extensive accumulation of dead bacteria. Notably, regardless of whether MRSA or *E. coli* formed the biofilms, the SSE+NIR group exhibited the lowest viable bacterial count, accompanied by extensive biofilm destruction and detachment. This finding indicates the separation of the biofilm from the substrate. Consequently, the combination of SSE+NIR enhanced the antibiofilm efficacy of the Bi_2_WO_6_:Yb,Er@CuS@CS nanoplatform.

The morphological changes of *E. coli* and MRSA bacteria following various interventions were analyzed using SEM. As illustrated in Figure 3f, both types of untreated bacteria remain viable, exhibiting typical cocci and bacilli morphology with a smooth surface. In the Dark group, SSE group, and NIR group, both types of bacteria exhibited varying degrees of distortion in their original morphology, along with the presence of wrinkles in the bacterial cell walls/membranes (indicated by red arrows). Notably, in the SSE+NIR group, the bacterial distribution was sparser, with a substantial number of bacteria exhibiting membrane wrinkling. Furthermore, a considerable portion of the bacteria experienced irreversible damage, including deformation, perforation, and fragmentation (indicated by red arrows). The results suggest that exposure to simulated sunlight alone or gentle near‐infrared radiation can lead to changes in bacterial cell membrane structure; however, these interventions are unlikely to produce destruction, resulting in limited antibacterial activity. Conversely, when these two methods are combined, they can cause more extensive damage to the bacterial cell membrane structure.

### 2.3 “Mild Thermal Therapy” Regulated the Polarization State of Macrophages

In the treatment of infected skin wounds, immune cells (particularly macrophages) play a pivotal role, where sustained M1 macrophage activity is crucial for long‐term antibacterial efficacy. However, persistent inflammatory states can impede skin healing, making the modulation of prolonged inflammation and timely promotion of M2 macrophage polarization essential for postinfection tissue repair [30, 57, 58].

**FIGURE 4:**
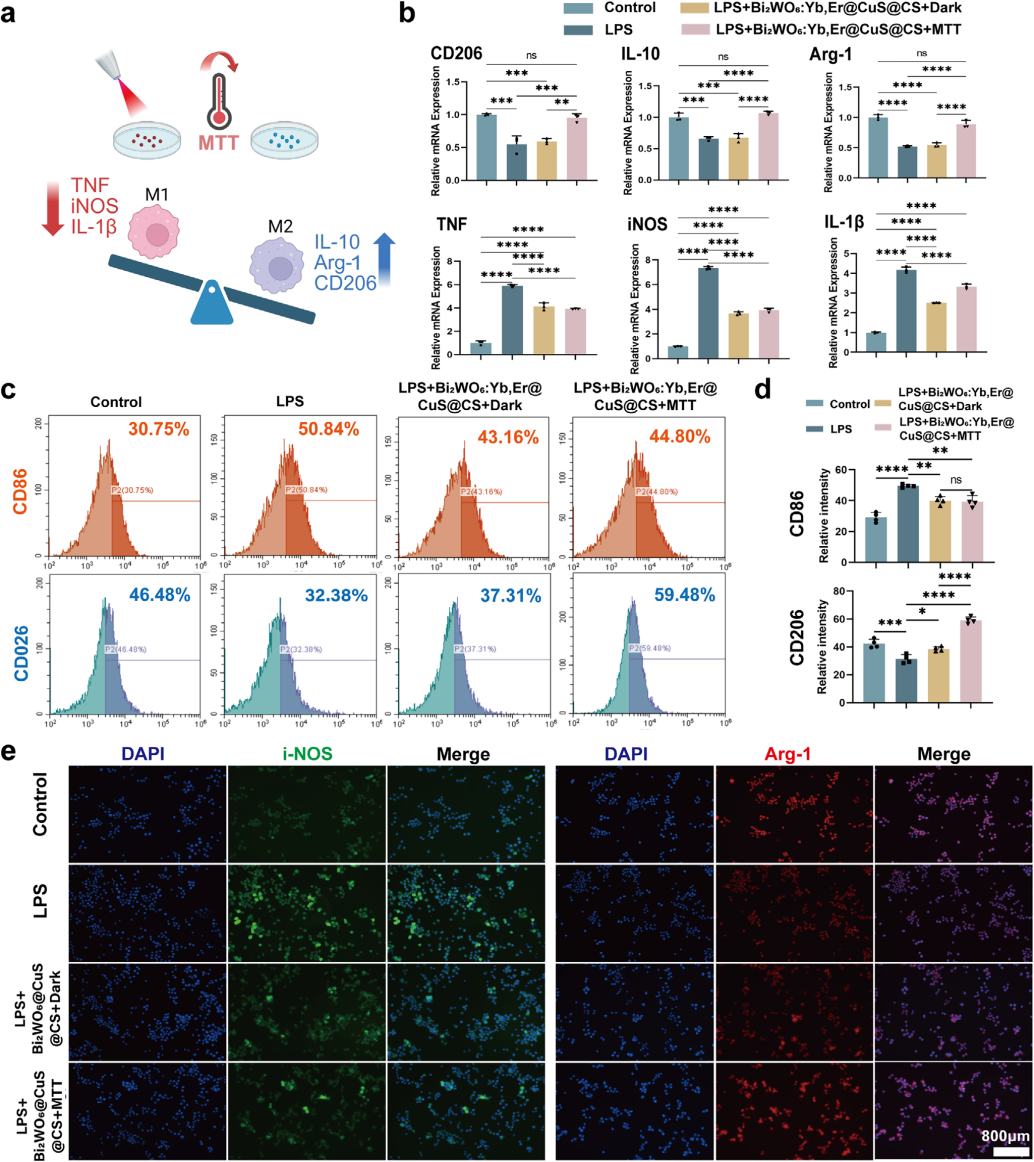
The immunomodulatory effects of the Bi_2_WO_6_:Yb,Er@CuS@CS nanoplatform on macrophages. (a) Schematic illustration of the immunomodulatory mechanism of “mild thermal therapy” (MTT) on macrophages. (b) mRNA expression levels of M1 macrophage markers (TNF‐α, iNOS, and IL‐1β) and M2 macrophage markers (CD206, IL‐10, and Arg‐1), *n* = 3. (c) Flow cytometry analysis of CD86 and CD206 expression in different treatment groups, with quantitative results shown in (d), *n* = 4. (e) Representative confocal microscopy images of Raw 264.7 cells under various treatments: iNOS (green, M1 phenotype), Arg‐1 (red, M2 phenotype). (ns, *p* > 0.05; *, *p* < 0.05; **, *p* < 0.01; ***, *p* < 0.001; ****, *p* < 0.0001).

In this work, cells were cocultured with Bi_2_WO_6_:Yb,Er@CuS@CS nanoplatforms and subjected to NIR irradiation (980 nm). Under thermal imaging guidance, the temperature was maintained at 42 ± 0.5°C for 15 min to achieve mild thermotherapy. The therapeutic regimen of mild thermal therapy (MTT) is illustrated in Figure 4a. To evaluate the early immunomodulatory effects of mild thermotherapy on infected skin wounds, macrophage phenotypic alterations were assessed via reverse transcription quantitative polymerase chain reaction (RT‐qPCR) one day post‐treatment. Initially, lipopolysaccharide (LPS) was used as an inducer to stimulate macrophages and establish an in vitro inflammatory model. As shown in Figure 4b, compared to the Control group, the LPS‐induced group exhibited significantly elevated expression of M1‐related markers (TNF, iNOS, IL‐1β) alongside reduced levels of M2‐related markers (CD206, IL‐10, Arg‐1), confirming successful establishment of the inflammatory model. Subsequently, the Bi_2_WO_6_:Yb,Er@CuS@CS nanoplatform was cocultured with inflammation‐activated macrophages under two distinct intervention conditions: a Dark group (no photothermal stimulation) and an MTT group. As demonstrated, both the Dark and MTT groups exhibited significant downregulation of M1‐related markers (TNF, iNOS, IL‐1β) compared to the LPS group after 24‐h coculture. The observed downregulation in the Dark group may be attributed to the immunomodulatory effects of Cu^2+^ on macrophage polarization. However, divergent outcomes were observed in M2 marker expression: while the Dark group showed no difference from the LPS group, mild thermotherapy significantly upregulated M2‐related markers (CD206, IL‐10, Arg‐1), restoring their expression to near‐Control levels. Subsequently, flow cytometry was used to evaluate the surface markers of macrophages following mild thermotherapy. Antigen‐presenting cell (APC)‐labeled CD86 was utilized to identify M1 macrophages, while phycoerythrin (PE)‐labeled CD206 served as an M2 macrophage marker. As shown in Figure 4c,d, consistent with RT‐qPCR results, LPS‐stimulated macrophages exhibited elevated CD86 expression and reduced CD206 expression compared to the Control group. In comparison to the LPS group, both the Dark and MTT groups showed comparable reductions in CD86 expression. However, the MTT group displayed significantly enhanced CD206 in the LPS group.

Furthermore, the immunomodulatory potential of mild thermotherapy was validated through immunofluorescence staining of alternative polarization markers: inducible nitric oxide synthase (i‐NOS, M1 marker) and arginase‐1 (Arg‐1, M2 marker). As shown in Figure 4e, LPS‐treated macrophages exhibited intensified i‐NOS (green) fluorescence and diminished Arg‐1 (red) signals relative to the Control group. Both intervention groups (Dark and mild thermotherapy) showed attenuated i‐NOS expression compared to LPS stimulation. Notably, only the MTT group manifested substantial upregulation of Arg‐1, corroborating prior experimental observations. Additionally, through further experiments, we have distinguished the roles of Cu^2+^ release and MTT in immunomodulatory. By comparing the RT‐qPCR and immunofluorescence staining results of the CuS@CS+Dark group and the CuS@CS+MTT group (Figure S7), it can be concluded that in the LPS‐induced inflammation model, Cu^2+^ can suppress the expression of proinflammatory (M1) macrophages and promote the expression of anti‐inflammatory (M2) macrophages. The introduction of MTT further enhances this effect.

These collective findings demonstrate that, in an in vitro inflammatory model, MTT effectively modulates macrophage polarization by suppressing proinflammatory M1 phenotypes while promoting anti‐inflammatory M2 differentiation. Given the established role of M2‐polarized macrophages in facilitating angiogenesis during wound healing, this immunomodulatory strategy aligns with the therapeutic objective of transitioning from infection control to tissue repair in infected skin wounds.

### 2.4 Transcriptomic Profiling of Macrophage Modulation by Mild Thermotherapy

Previous studies have demonstrated that mild magnetic hyperthermia promotes M1‐to‐M2 macrophage polarization in infected bone defects via the PI3K–Akt signaling pathway [27]. However, the precise immunomodulatory mechanisms of mild thermotherapy (MTT) in infected skin wounds healing remain unclear and warrant further investigation.

**FIGURE 5:**
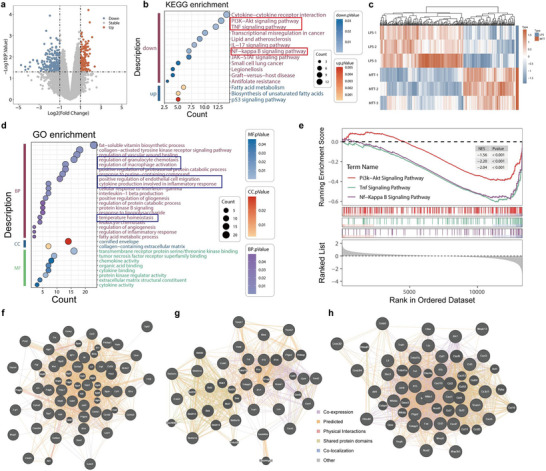
Transcriptomic analysis of the anti‐inflammatory effects induced by “mild thermal therapy” (MTT). (a) Volcano plot of transcriptome data from LPS‐pretreated Raw 264.7 cells after MTT treatment (*Q* < 0.05). (b) KEGG enrichment analysis of potential genes involved in identified pathways and processes in LPS‐pretreated Raw 264.7 cells after MTT treatment. (c) Heatmap analysis of pathway‐related genes comparing LPS‐pretreated Raw 264.7 cells with MTT‐treated LPS‐pretreated Raw 264.7 cells. (d) GO enrichment analysis of biological processes potentially associated with MTT‐treated LPS‐pretreated Raw 264.7 cells. (e) GSEA analysis of immune pathways regulated by MTT. (f–h) Protein–protein interaction networks within the (f) PI3K–Akt, (g) TNF, and (h) NF–κB signaling pathways.

Microarray analysis was performed to compare mRNA expression profiles in LPS‐primed macrophages with or without MTT treatment. Volcano plot analysis (Figure 5a) revealed that MTT significantly upregulated 125 genes and downregulated 220 genes (*Q* < 0.05) compared to LPS treatment alone. Kyoto Encyclopedia of Genes and Genomes (KEGG) pathway enrichment analysis (Figure 5b) identified several inflammation‐related pathways, including PI3K–Akt signaling, TNF signaling, and NF–κB signaling (*Q* < 0.05), suggesting their involvement in the anti‐inflammatory effects of MTT. Hierarchical clustering heatmap (Figure 5c) demonstrated strong intra‐ and intergroup concordance in gene expression patterns, indicating consistent transcriptional reprogramming in MTT‐treated macrophages. Gene ontology (GO) enrichment analysis (Figure 5d) further elucidated the biological processes associated with MTT‐mediated immunomodulation, particularly in the regulation of inflammatory cell activation, cytokine production, and endothelial cell migration. Gene set enrichment analysis (GSEA) of the PI3K–Akt, TNF, and NF–κB signaling pathways (Figure 5e) revealed significant downregulation (*p* < 0.01) in MTT‐treated macrophages compared to LPS‐primed controls. These pathways form an interconnected “Inflammatory Network,” where TNF initiates the response, NF–κB amplifies inflammatory signals, and PI3K–Akt regulates inflammation duration and intensity by modulating cell survival and cross‐talk with other pathways. Thus, suppression of these pathways represents a key mechanism underlying MTT's anti‐inflammatory effects. A protein–protein interaction network (Figure 5f–h) was constructed using core genes from the PI3K–Akt, TNF, and NF–κB pathways identified in GSEA, further highlighting their functional interplay in macrophage polarization.

### 2.5 In Vitro Promotion of Angiogenesis of Bi_2_WO_6_:Yb,Er@CuS@CS

Angiogenesis is a critical aspect of tissue repair, as the restoration of blood flow to damaged tissues provides essential oxygen and nutrients to support the growth and function of reparative cells. However, angiogenesis is also a highly complex process involving intricate interactions among endothelial cells, pericytes, and immune cells. Numerous studies have demonstrated that M2‐polarized macrophages play a pivotal role in tissue repair and wound healing by secreting proangiogenic factors [59, 60, 61]. Thus, immunomodulation to promote angiogenesis represents a promising therapeutic strategy. In the context of infected skin wounds, the final step of tissue regeneration relies on efficient vascularization, which significantly accelerates the overall healing process.

**FIGURE 6:**
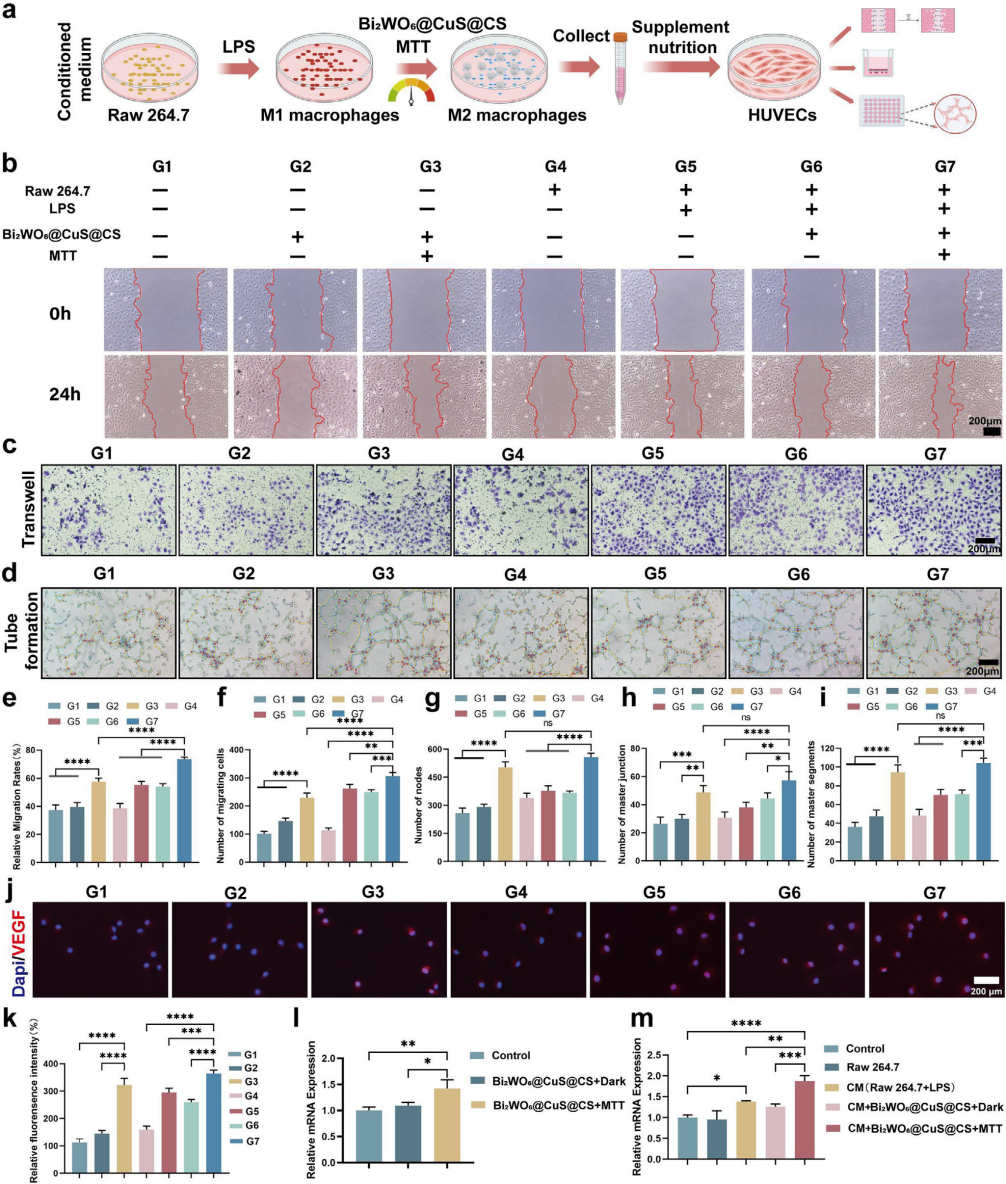
Direct effects of the Bi_2_WO_6_:Yb,Er@CuS@CS nanoplatform on in vitro angiogenesis and MTT‐induced immunomodulatory effects on angiogenesis. (a) Schematic diagram illustrating the effects of conditioned media from different treatments on HUVECs migration and tube formation. (b–d) Images of scratch assay (b), Transwell migration assay (c), and tube formation assay (d) of HUVECs treated with conditioned media from different groups. (e) Relative migration rate of HUVECs in scratch assays, *n* = 3. (f) Number of migrated HUVECs in Transwell assays, *n* = 3. (g–i) Quantitative analysis of node numbers (g), master junction numbers (h), and master segment numbers (i) in tube formation assays. (j) Representative confocal microscopy images of VEGF expression in HUVECs under different treatments, VEGF (red), DAPI (blue): nuclei. (k) Quantitative analysis of VEGF immunofluorescence intensity, *n* = 3. (l) mRNA expression of VEGF in HUVECs under different treatments, *n* = 3. (m) mRNA expression of VEGF in HUVECs treated with conditioned media from different groups, *n* = 3.(ns, *p* > 0.05; *, *p* < 0.05; **, *p* < 0.01; ***, *p* < 0.001; ****, *p* < 0.0001).

To evaluate the proangiogenic potential of the Bi_2_WO_6_:Yb,Er@CuS@CS nanoplatform, we employed a dual‐approach experimental design: Direct coculture of the nanoplatform with HUVECs under varying treatment conditions and conditioned medium (CM) assay, in which HUVECs were cultured with macrophage‐derived CM following different interventions. The preparation of conditioned media is illustrated in Figure 6a. In vitro migration and angiogenic assays were performed using scratch assays and Transwell migration assays to assess HUVEC motility. As shown in Figure 6b–f, in G1–G3 (direct coculture groups), G3 (nanoplatform+MTT) exhibited the strongest migratory capacity, with the highest wound closure rate and Transwell‐migrated cell count, suggesting that Cu^2+^ release under mild hyperthermia (42 ± 0.5°C) may enhance endothelial migration. In G4–G7 (CM‐treated groups), G5 (Raw264.7+LPS) and G6 (Raw264.7+LPS+nanoplatform) showed improved migration compared to G4 (Raw264.7 alone), likely due to the combined effects of LPS and nanoplatform‐induced immunomodulation. Notably, G7 (Raw264.7+LPS+nanoplatform+MTT) demonstrated the highest migration rate and cell count, indicating that MTT‐enhanced macrophage conditioning maximizes the secretion of promigratory cytokines. Matrigel tube formation assay further confirmed these findings. As shown in Figure 6d, G7‐treated HUVECs exhibited enhanced tubular network formation, characterized by tight intercellular junctions, branching nodes, and capillary‐like structures, all of which are hallmarks of robust angiogenesis. Quantitative analysis of node numbers (Figure 6g), master junction numbers (Figure 6h), and master segment numbers (Figure 6i) corroborated these observations. Vascular endothelial growth factor (VEGF), one of the most potent angiogenic factors in skin, critically influences wound healing. Immunofluorescence staining (Figure 6j,k) revealed that G7 displayed the highest VEGF expression (red fluorescence intensity), followed by G3, G5, and G6. RT‐qPCR (Figure 6l,m) further validated these results, with G7 showing the most significant upregulation of VEGF mRNA.

These findings demonstrate that the Bi_2_WO_6_:Yb,Er@CuS@CS nanoplatform promotes angiogenesis through dual mechanisms. Direct stimulation of HUVECs migration and tube formation via Cu^2+^‐mediated and MTT‐enhanced effects. Indirect immunomodulation by polarizing macrophages toward a proangiogenic phenotype, thereby enriching the CM with VEGF and other angiogenic cytokines. The synergistic combination of MTT and immunomodulation not only enhances endothelial function but also establishes a proregenerative microenvironment, positioning this nanoplatform as a promising candidate for accelerated wound healing and the repair of vascularized tissue.

### 2.6 In Vivo Promotion of Infected Skin Wounds Healing by Bi_2_WO_6_:Yb,Er@CuS@CS

The experimental results demonstrate that the Bi_2_WO_6_:Yb,Er@CuS@CS nanoplatform exhibits excellent biosafety, enhanced antibacterial activity, immunomodulatory capability, and proangiogenic effects‐all crucial properties for repairing infected skin wounds. To evaluate its in vivo wound healing efficacy, we established a full‐thickness MRSA‐infected skin wound model on the dorsum of C57BL/6 mice.

**FIGURE 7:**
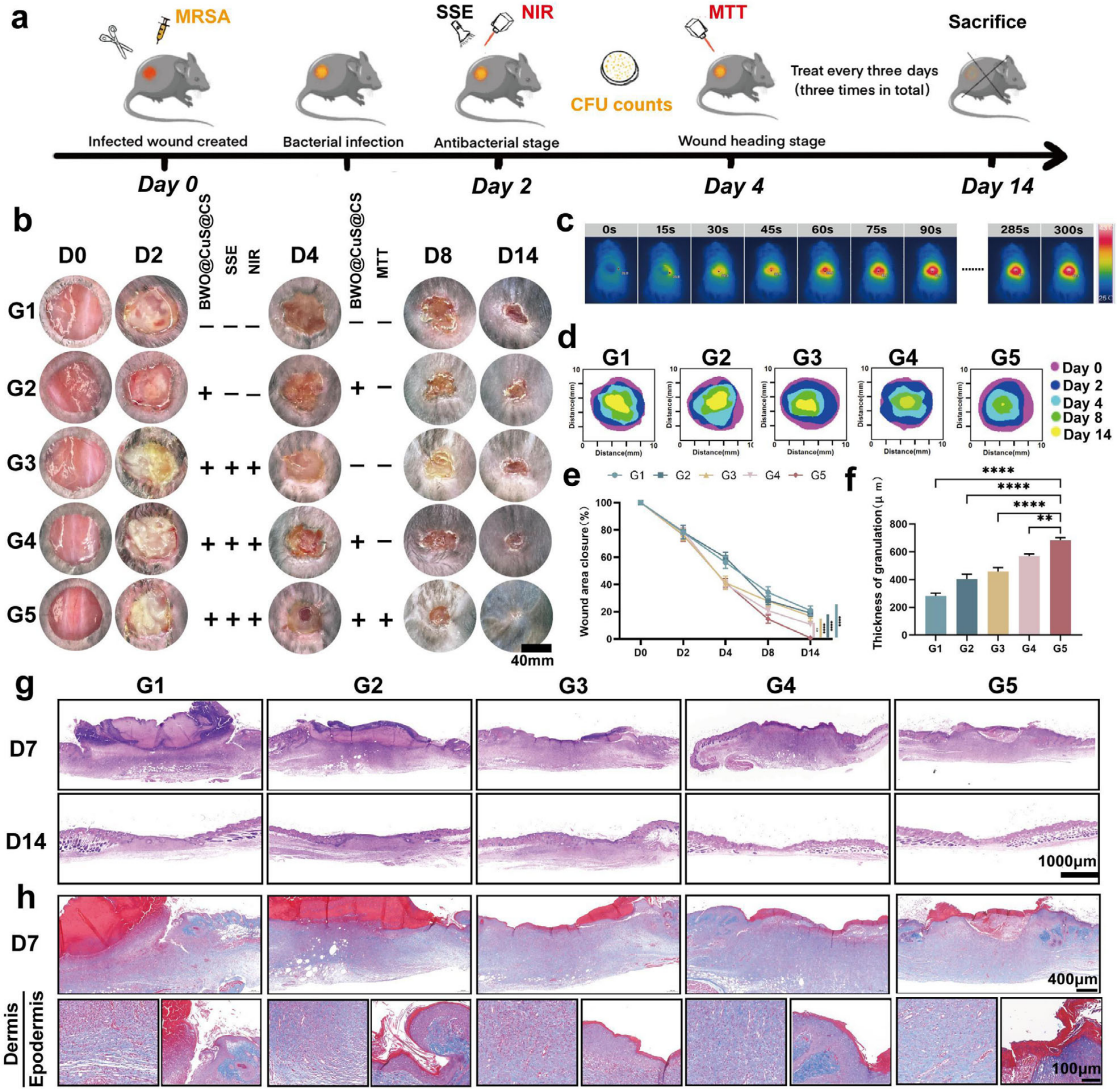
Bi_2_WO_6_:Yb,Er@CuS@CS nanoplatform promotes infected wound healing in vivo. (a) Timeline of infected skin wounds modeling and therapeutic regimen in mice. (b) Representative photographs of wound sites on Days 0, 2, 4, 8, and 14 postmodeling with annotated treatment measures. (c) Representative infrared thermal images during MTT treatment. (d) Schematic diagram of wound healing progression. (e) Wound closure rates across different groups, *n* = 3. (f) Quantitative analysis of granulation tissue thickness in samples harvested on Day 7, *n* = 3. (g) H&E staining of infected wound tissues on Day 7 and Day 14. (h) Masson's staining images of collagen fibers in cross‐sectional wound tissues harvested on Day 7. (ns, *p* > 0.05; *, *p* < 0.05; **, *p* < 0.01; ***, *p* < 0.001; ****, *p* < 0.0001).

As illustrated in the schematic diagram (Figure 7a), the animal experiment was divided into two phases: an early anti‐infection phase (Days 0–4) and a later wound healing phase (Days 4–14). Macroscopic wound images (Figure 7b), schematic diagram of wound healing progression (Figure 7d), and wound closure rates (Figure 7e) at different time points (Days 0, 2, 4, 8, and 14) are shown. During MTT treatment, the process was monitored using an infrared thermal imaging camera (Figure 7c). On Day 2, all groups exhibited yellow purulent exudate. By Day 4, groups G3, G4, and G5, which received complete antibacterial treatment, showed better healing outcomes, with wound sizes of 51.8%, 55.3%, 36.0%, 39.7%, and 37.5% for G1–G5, respectively. On Day 14, the infected wounds in G5 were nearly completely healed and covered with newly formed skin. In contrast, G1 and G2 wounds remained covered with eschar, while G3 and G4 showed faster healing than these two groups. Notably, G4 demonstrated better healing than G3 due to regular Bi_2_WO_6_:Yb,Er@CuS@CS treatment during the healing phase. Hematoxylin and eosin (H&E) staining was used to analyze the histological status during wound healing. Granulation tissue, primarily composed of capillaries and fibroblasts, plays a key role in postinjury tissue repair. As shown in Figure 7f,g, on Day 7, G1–G4 exhibited substantial scar tissue with minimal new tissue formation, indicating minor differences among these groups. In contrast, G5 displayed abundant granulation tissue with significantly reduced scar formation, indicating superior healing efficiency. By Day 14, while all groups showed accelerated wound healing, the length of granulation tissue varied considerably (Figure S8). G1 had the widest granulation tissue among all groups, while G5 showed the narrowest granulation tissue with minimal scarring, suggesting optimal wound healing in this group. Masson's staining revealed collagen deposition and epidermal thickness in the wounds. On postoperative Day 7 (Figure 7h and Figure S3a), G5 exhibited the highest amount of newly formed collagen (blue). The epidermal thickness (Figure S9) at the wound center was also most significant in G5, reaching 29.2 ± 2.25 µm. These results collectively demonstrate that the Bi_2_WO_6_:Yb,Er@CuS@CS nanoplatform can effectively promote the healing of MRSA‐infected wounds.

### 2.7 In Vivo Therapeutic Evaluation of Bi_2_WO_6_:Yb,Er@CuS@CS

To evaluate the in vivo antibacterial efficacy of the Bi_2_WO_6_:Yb,Er@CuS@CS nanoplatform, wound tissue homogenates from Day 4 post‐treatment were serially diluted and plated on blood agar plates. As shown in Figure S10, after 24 h of incubation, the complete antibacterial treatment groups (G3–G5) demonstrated significantly lower bacterial colony counts compared to both the control group (G1) and the nanoplatform‐only group (G2), indicating potent in vivo anti‐infective activity.

**FIGURE 8:**
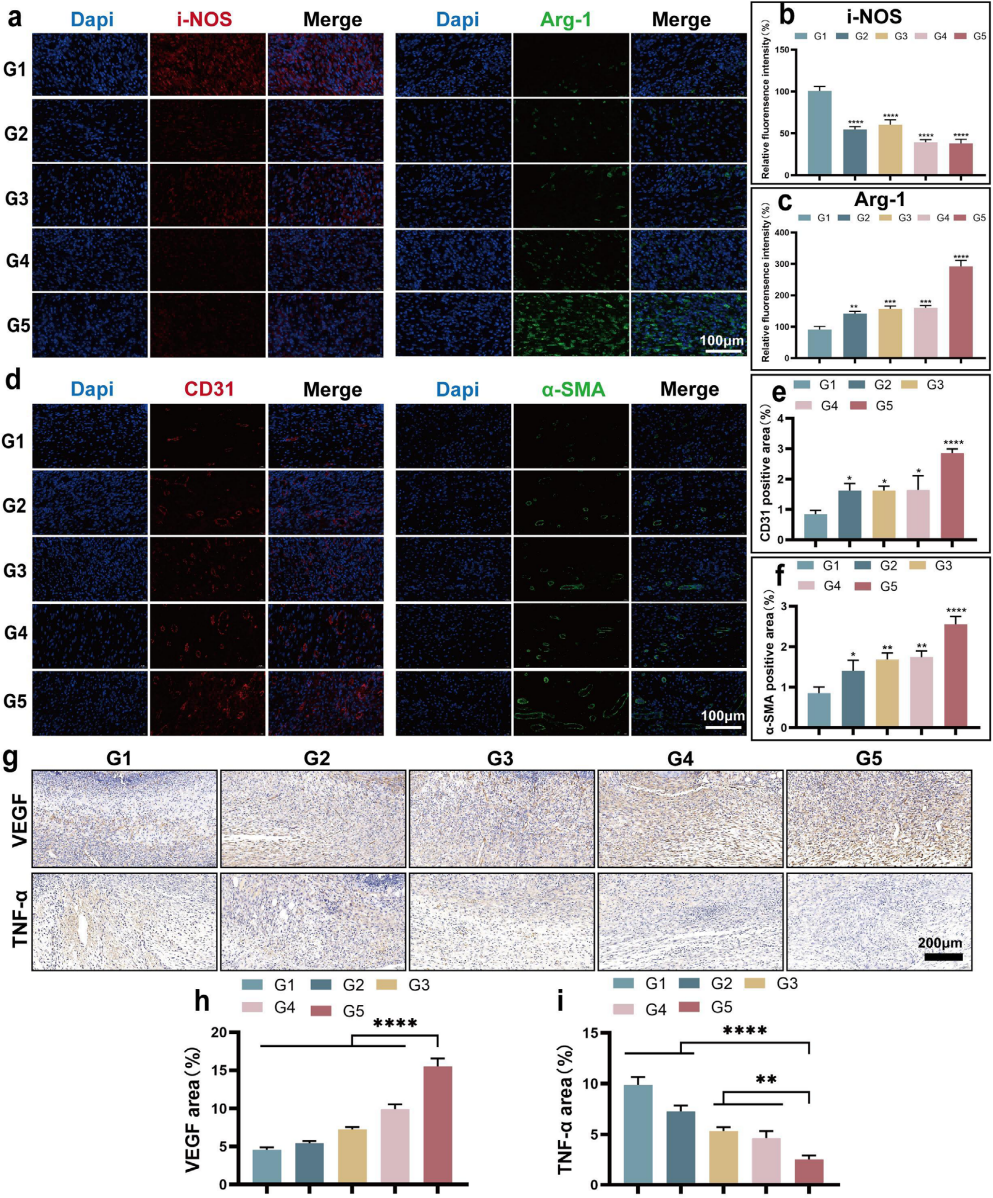
Bi_2_WO_6_:Yb,Er@CuS@CS nanoplatform suppresses inflammatory microenvironment and promotes angiogenesis in vivo. (a) Representative immunofluorescence images of iNOS (M1 marker) and Arg‐1 (M2 marker) stained sections from different groups on Day 7. (b) Quantitative analysis of iNOS fluorescence intensity and (c) Arg‐1 fluorescence intensity, *n* = 3. (d) Representative immunofluorescence images of CD31 and α‐SMA stained sections from different groups on Day 14. (e) Quantitative analysis of CD31‐positive area and (f) α‐SMA‐positive area, *n* = 3. (g) Immunohistochemical staining images of VEGF and proinflammatory cytokine TNF‐α in wound granulation tissues on Day 7. (h) Quantitative analysis of VEGF‐positive area and (i) TNF‐α‐positive area. (j) The explanation of groups. (ns, *p* > 0.05; *, *p* < 0.05; **, *p* < 0.01; ***, *p* < 0.001; ****, *p* < 0.0001).

To investigate the effect of Bi_2_WO_6_:Yb,Er@CuS@CS‐mediated MTT on macrophage polarization in vivo, we performed immunofluorescence staining for iNOS (red, M1 marker) and Arg‐1 (green, M2 marker). As shown in Figure 8a–c, on Day 7, the G5 group exhibited extensive M2 macrophage infiltration, with a significantly higher M2/M1 ratio compared to other groups. This indicates an early shift from the inflammatory phase to the repair phase, supported by quantitative analysis. Furthermore, the expression level of tumor necrosis factor‐α (TNF‐α) was evaluated through immunohistochemical analysis, which similarly supported the results mentioned above. TNF‐α plays a critical role in inflammatory responses. As shown in Figure 8g,i, the G5 group exhibited the lowest TNF‐α expression on Day 7, indicating minimal inflammatory activity. Thus, the Bi_2_WO_6_:Yb,Er@CuS@CS nanoplatform, as demonstrated by MTT, effectively regulates the local immune microenvironment, playing a pivotal role in wound healing.

Moreover, during the healing phase of infected wounds, the activity of angiogenesis significantly influences the rate of wound recovery. As shown in Figure 8d–f, G5 demonstrated higher vascular density during the late healing stage, as supported by CD31 (red) and α‐SMA (green) immunofluorescence staining and quantitative analysis. CD31 is typically expressed in vascular endothelial cells, while α‐SMA serves as a marker for myofibroblasts, both being valuable indicators for assessing angiogenesis levels. VEGF, a key mediator of angiogenesis, is weakly expressed in normal skin tissues, primarily in fibroblasts and epidermal cells. However, its expression increases significantly in newly formed skin tissue after injury. As demonstrated in the immunohistochemical results of Figure 8g,h, on Day 14, the G5 group showed the highest percentage of VEGF‐positive area, indicating enhanced VEGF expression and accelerated angiogenesis compared to the other groups. The controlled inflammatory response and improved vascularization imply that the Bi_2_WO_6_:Yb,Er@CuS@CS nanoplatform, combined with mild photothermal therapy (MTT), modulates macrophage polarization, thereby influencing immune regulation. During wound healing, macrophages transition from a proinflammatory M1 phenotype (essential for antibacterial defense) to an anti‐inflammatory M2 phenotype (critical for tissue repair). Persistent inflammation delays healing, making the timely resolution of inflammation crucial in infected wounds. Based on these findings, the superior wound‐healing efficacy of the Bi_2_WO_6_:Yb,Er@CuS@CS nanoplatform can be attributed to two key mechanisms: (1) potent antibacterial activity, reducing bacterial load and associated inflammation; and 2) immunomodulation via M2 macrophage polarization, promoting timely transition to the repair phase.

Finally, to confirm biosafety, major organs were harvested at the study endpoint and subjected to H&E staining (Figure S11). No significant histopathological abnormalities were observed, confirming the biocompatibility of the Bi_2_WO_6_:Yb,Er@CuS@CS nanoplatform. These findings highlight its potential as a safe and effective therapeutic agent for wound repair in infected wounds.

## 3 Conclusion

In summary, we have developed a biocompatible and multifunctional Bi_2_WO_6_:Yb,Er@CuS@CS nanoplatform for the “triple‐action” therapy of infected skin wounds. The optimized integration of Bi_2_WO_6_:Yb,Er, CuS, and CS endows the nanoplatform with dual‐photoexcitation capability, enabling synergistic antibacterial effects through PDT, PTT, and metal ion release. This combined approach achieves inhibition rates of over 99% against both MRSA and *E. coli*, demonstrating significantly enhanced antibacterial activity. Moreover, mild photothermal treatment with this nanoplatform modulates macrophage polarization, promoting angiogenesis and accelerating wound healing. In vivo experiments using MRSA‐infected wound models demonstrated that the Bi_2_WO_6_:Yb,Er@CuS@CS nanoplatform exhibits excellent anti‐infective and immunomodulatory properties, facilitating granulation tissue formation, collagen deposition, and neovascularization, thereby markedly improving the healing process of infected wounds. Transcriptomic analysis further revealed that the nanoplatform‐mediated MTT regulates the immune microenvironment by downregulating key signaling pathways, including the PI3K–Akt, TNF, and NF–κB pathways. Despite the structural complexity of the BWO:Yb,Er@SiO_2_@CuS system, the present design serves as a modular framework for multifunctional antibacterial nanoplatforms. Future studies may focus on simplifying the architecture by optimizing component composition to reduce cost, while systematically elucidating the contribution of each functional module. Such efforts will enable the rational development of cost‐effective and mechanism‐defined antibacterial materials. Collectively, this study highlights the Bi_2_WO_6_:Yb,Er@CuS@CS nanoplatform as a promising therapeutic candidate for the repair of infected skin wounds, offering a comprehensive approach, which combines antibacterial action, immunomodulation, and tissue regeneration.

## 4 Experimental Section

### 4.1 Chemicals

Bismuth trichloride (BiCl_3_), ytterbium(III) chloride hexahydrate (YbCl_3_·6H_2_O), erbium(III) chloride hexahydrate (ErCl_3_·6H_2_O), sodium tungstate (Na_2_WO_4_), methylene blue (MB), and PEG‐2000 were purchased from Sigma‐Aldrich. Dimethyl sulfoxide (DMSO), ammonium hydroxide (NH_3_·H_2_O), tetraethylorthosilicate (TEOS), (3‐aminopropyl)triethoxysilane (APTES), sodium citrate (Na_3_Cit), copper(II) chloride dihydrate (CuCl_2_·2H_2_O), and sodium sulfide nonahydrate (Na_2_S·9H_2_O) were purchased from Aladdin. MES buffer, chitosan, 1‐(3‐dimethylaminopropyl)‐3‐ethylcarbodiimide hydrochloride (EDC·HCl), *N*‐hydroxysuccinimidh (NHS), and ethanol were purchased from Macklin. All materials were used without additional purification.

### 4.2 Preparation of Bi_2_WO_6_:Yb,Er Nanoparticles

A total of 1 mmol of BiCl_3_, YbCl_3_·6H_2_O, and ErCl_3_·6H_2_O was dispersed in 8 mL of deionized water. Under magnetic stirring, a nitric acid solution was added dropwise until the solution became clear, followed by the addition of 0.06 g of PEG‐2000 to form Solution A. Meanwhile, 0.5 mmol of Na_2_WO_4_ was dispersed in 25 mL of deionized water to form Solution B. Under magnetic stirring, Solution B was added dropwise to Solution A to create a mixed solution. The pH of the mixture was adjusted to 11 using a NaOH solution, and stirring was continued for 15 min. The mixed solution was transferred to a 50 mL Teflon‐lined stainless‐steel autoclave and heated to 180°C for 4 h before being cooled to room temperature. The precipitate was collected by centrifugation at 10,000 rpm for 5 min, washed three times with ethanol and water, and dried in an oven at 60°C.

### 4.3 Preparation of Bi_2_WO_6_:Yb,Er@SiO2 Nanoparticles

50 mg of Bi_2_WO_6_:Yb,Er powder was dispersed in 10 mL of DMSO, followed by sonication for 30 min and subsequent stirring for another 30 min. 600 µL of NH_3_·H_2_O and 150 µL of TEOS were added dropwise to the mixture, which was then stirred overnight. 200 µL of APTES was introduced into the mixed solution and stirred for 5 h. The precipitate was obtained by centrifugation at 10,000 rpm for 5 min, washed twice with ethanol, and dried in an oven at 60°C.

### 4.4 Preparation of CuS Nanoparticles

0.014 g of CuCl_2_·2H_2_O and 0.0179 g of Na_3_Cit were dissolved in 100 mL of deionized water. 0.0192 g of Na_2_S·9H_2_O was dissolved in 2 mL of deionized water. The Na_2_S solution was added dropwise to the mixture of CuCl_2_ and Na_3_Cit, and the mixture was stirred for 5 min. The mixture was then stirred in a 90°C water bath for 15 min, during which the solution turned dark green. After the reaction, the mixture was immediately placed in an ice‐water bath and stored at 4°C to prevent further reaction.

### 4.5 Preparation of Bi_2_WO_6_:Yb,Er@SiO_2_@CuS@Chitosan Nanoplatforms

Bi_2_WO_6_:Yb,Er@SiO_2_ powder was mixed with 20 mL of CuS solution and stirred for 2 h. The precipitate was obtained by centrifugation at 10,000 rpm for 5 min, washed twice with deionized water, and dispersed in 20 mL of deionized water. The resulting Bi_2_WO_6_:Yb,Er@SiO_2_@CuS dispersion was mixed with 0.1 g of succinic acid and stirred for 12 h. The precipitate was then collected by centrifugation at 10,000 rpm for 5 min, washed twice with deionized water, and dispersed in 50 mL of 0.1 mol L^−1^ MES buffer solution (pH = 4.7). Subsequently, 0.25 g of chitosan was added to the mixture, and a homogeneous gel solution was formed after sonication for 1 h. Then, 0.326 g of EDC·HCl and 0.391 g of NHS were introduced, and the reaction was stirred under light‐protected conditions for 24 h. Finally, the precipitate was isolated by centrifugation at 10,000 rpm for 5 min, washed twice with MES buffer solution, and redispersed in MES buffer (pH = 4.7) for further use.

### 4.6 Characterization

TEM images were acquired using a Thermofisher Talos L120C G2 electron microscope at 120 kV. HRTEM and EDS mapping were acquired using a Talos F200X G2 electron microscope. XRD patterns were recorded on a Bruker D8 Advance diffractometer with Cu Kα radiation. UV–vis absorption and fluorescence spectra were measured at room temperature using an FLS‐1000 spectrophotometer (Edinburgh Instruments) and an RF‐6000 fluorescence spectrophotometer. XPS analyses were performed on a Thermofisher ESCALAB QXi spectrometer. Photothermal experiments were conducted using a 980 nm laser source generated by a Laser Diode Controller (SFOLT Co. Ltd., China). Thermal images were recorded using a thermal imaging camera (FTIR, USA). ICP‐OES results were measured using an Avio 500 inductively coupled plasma optical emission spectrometer.

### 4.7 Photodynamic Degradation Experiment of MB

Photodynamic activities of PBS, pure Bi_2_WO_6_ and Bi_2_WO_6_:Yb,Er were investigated by degradation of MB under white light irradiation. First, the pure Bi_2_WO_6_ and Bi_2_WO_6_:Yb,Er nanoparticles (50 mg) were ultrasound‐dispersed in PBS solution of MB (50 mL, 0.15 mm). Then, the mixture of powders and dye solution was magnetically stirred in the dark for 1 h to achieve an absorption–desorption equilibrium. Afterward, 3 mL of suspension was taken and centrifuged. The concentration of MB was determined by measuring the UV–vis absorption based on the absorbance band of 660 nm.

### 4.8 Photothermal Experiment

Photothermal properties of PBS, CuS, pure Bi_2_WO_6_, and Bi_2_WO_6_:Yb,Er were investigated under 980 nm laser irradiation. 1 mL of aqueously dispersed photothermal agents at different concentrations was added in a quartz cuvette, followed by irradiation with a 980 nm laser (1 W/cm^2^, 6 min). An FTIR E50 camera was used to record both the thermal images and temperature variation data during irradiation.

### 4.9 Photothermal Conversion Efficiency Experiments

1 mg/mL Bi_2_WO_6_:Yb,Er@CuS@CS in 2 mL PBS buffer was dropped in impending 4 mL transparent plastic centrifugal tube. Then the solutions were exposed to 980 nm light at the power density of 1 W/cm^2^ for 300 s and simultaneously imaged by an IR thermal camera (FLIR, USA). The photothermal conversion efficiency (*η*) of Bi_2_WO_6_:Yb,Er@CuS@CS, was calculated using Equation (1):

(1) $$\eta =\frac{\mathrm{hS}\left(T_{\max}-T_{\mathrm{surr}}\right)-Q_{\mathrm{Dis}}}{I\left(1-10^{-\mathrm{As}}\right)}$$

where *I* represents the laser power, As is the absorbance of nanoplatforms at 980 nm, *T*_max_ is the equilibrium temperature, *T*_surr_ is the ambient temperature of the surroundings, *h* is heating transfer coefficient, and *S* is the surface area of the tube. These parameters are determined using Equation (2):

(2) $$\tau_{s}=\frac{mc}{hS}$$

where *m* is the mass of the solution, *c* is the specific heat capacity of the solvent, and *τ*_s_ is a time constant, which can be determined in Equation (3):

(3) $$t=-\tau_{s}\ln(\theta )$$

*θ* is a time‐dependent dimensionless parameter, defined in Equation (4):

(4) $$\theta =\frac{T-T_{\mathrm{surr}}}{T_{\max}-T_{\mathrm{surr}}}$$

*Q*_dis_ is the light absorption of the quartz tube, which can be determined using Equation (5):

(5) $$Q_{\mathrm{Dis}}=\frac{\left(T_{\max}-T_{\mathrm{surr}}\right)\mathrm{mc}}{t}$$

### 4.10 Cell Culture

Raw264.7 macrophages (Raw 264.7) and HUVECs were obtained from the Cell Bank of the Chinese Academy of Science (Shanghai, China). Raw 264.7 was cultured in α‐MEM (Gibco, USA) supplemented with 1% penicillin–streptomycin (P/S; Gibco, USA) and 10% fetal bovine serum (Gibco, USA). HUVECs were cultured in an Endothelial Cell Medium (ScienCell, USA). All cells were maintained at 37°C in a humidified atmosphere with 5% CO_2_.

### 4.11 Biocompatibility Testing

HUVECs were seeded in 96‐well plates at a density of 5000 cells per well and divided into two groups. One group was only supplemented with different concentrations of Bi_2_WO_6_:Yb,Er@CuS@CS (0, 50, 100, 200, 400, and 600 µg/mL) and cocultured with HUVECs without any other intervention measures, the other group applied an additional complete intervention, which included 30 min of SSE and 15 min of 1.0 W/cm^2^, 980 nm NIR (NIR). After 1, 2, and 3 days of culture, 100 µL of culture medium containing 10% CCK‐8 reagent (Dojindo, Japan) was added to each well and incubated for 2 h. After this incubation, the absorbance at 450 nm was measured using a microplate reader. To evaluate cytotoxicity, live/dead staining was conducted in the second group. Live cells, stained with calcein‐AM (Beyotime Biotechnology, China), showed green fluorescence, while dead cells, stained with PI (Beyotime Biotechnology, China), showed red fluorescence. To verify the morphology of the cells, HUVECs were seeded in confocal dishes and divided into five groups: Control, Dark, SSE, NIR, and SSE+NIR—the group without Bi_2_WO_6_:Yb,Er@CuS@CS, named as the control group. The remaining groups contained 200 µg/mL Bi_2_WO_6_:Yb,Er@CuS@CS and were subjected to the following interventions: Dark): cultured in complete darkness throughout, SSE): irradiated with simulated sunlight exposure for 30 min, NIR): irradiated with 980 nm NIR (1.0 W/cm^2^) for 15 min; SSE+NIR): irradiated with simulated sunlight exposure for 30 min and 980 nm NIR (1.0 W/cm^2^) for 10 min. After 2 days of culture, HUVECs were stained with FITC‐phalloidin and DAPI (Beyotime Biotechnology, China) for immunofluorescence to visualize their morphology. The morphology of the cells was observed under a confocal microscope.

### 4.12 Preparation of Bacterial Suspension and Biofilm

We used MRSA and *E. coli* as experimental strains. MRSA (ATCC 43300) and *E. coli* (ATCC 25922) were obtained from the Department of Microbiology Laboratory, Shanghai Jiao Tong University Affiliated Sixth People's Hospital. Activated MRSA and *E. coli* were removed from the refrigerator at 4°C and inoculated onto tryptone soy agar (TSB). After overnight cultivation in a shaker at 37°C, the MRSA suspension and *E. coli* suspension were obtained. Biofilms of MRSA and *E. coli* were prepared in confocal dishes. After overnight cultivation in TSB containing 0.25% glucose, the bacteria were inoculated into the confocal dish at a concentration of 1×10^5^ CFU/mL. The confocal dishes were incubated at 37°C for 48 h without shaking, and biofilms of MRSA and *E. coli* were finally obtained.

### 4.13 In Vitro Antibacterial and Antibiofilm Activity Assays

The resuscitated MRSA and *E. coli* bacterial suspensions (10^7^ CFU/mL) were subjected to group‐specific interventions, with the Bi_2_WO_6_:Yb,Er@CuS@CS nanoplatform maintained at a concentration of 200 µg/mL across all treated groups. The experimental groups were designed as follows: Control group: PBS addition without any further treatment; Dark group: Nanoplatform addition followed by incubation under dark conditions; SSE group: Nanoplatform addition with 30 min of SSE; NIR group: Nanoplatform addition with 15 min of 1.0 W/cm^2^ near‐infrared (NIR, 980 nm) irradiation; SSE+NIR group: Nanoplatform addition combined with both 30 min of SSE and 15 min of NIR irradiation. Post‐treatment, the bacterial suspensions were serially diluted, and 100 µL aliquots were spread onto sheep blood agar (SBA) plates. These plates were incubated overnight at 37°C, after which bacterial colonies were enumerated to calculate antibacterial efficiency.

For biofilm assays, MRSA and *E. coli* biofilms were prepared on confocal dishes and titanium substrates using the methodology mentioned above. These biofilms underwent identical treatments (Control, Dark, SSE, NIR, and SSE+NIR). Biofilms in confocal dishes were fixed, stained with crystal violet, and quantified via absorbance measurement at 550 nm. For titanium‐adhered biofilms, post‐treatment staining was performed using PI and SYTO‐9, followed by imaging via confocal laser scanning microscopy. For SEM (Zeiss EVO 18, Germany), titanium‐supported biofilms were fixed, dehydrated, sputter‐coated with gold, and subsequently imaged.

### 4.14 In Vitro ROS Generation Assay

Raw 264.7 cells were seeded in 24‐well plates at a density of approximately 5×10^4^ cells/well and cultured for 24 h. The cells were then divided into the following treatment groups: control group, SSE group, Bi_2_WO_6_:Yb,Er@CuS@CS+Dark group, and Bi_2_WO_6_:Yb,Er@CuS@CS+SSE group. Subsequently, the fluorescent probe DCFH‐DA (Beyotime, China) at working concentration was added to each well, followed by incubation at 37°C for 20 min. The cells were washed three times with PBS to thoroughly remove any extracellular DCFH‐DA. Finally, images were captured using an inverted fluorescence microscope. Differentiation of ROS types was performed using three fluorescent probes: DHE (Beyotime, China), SOSG (Beyotime, China), and HPF (MCE, China). Similarly, Raw 264.7 cells were seeded in black 96‐well plates at approximately 3000 cells/well and cultured for 24 h before treatment. After intervention, different fluorescent dyes were added to distinguish superoxide anion (·O_2_^−^), hydroxyl radicals (·OH), and singlet oxygen (^1^O_2_). Following 15 min of incubation at 37°C in the dark, fluorescence was measured using a Fluorescence Microplate Reader.

### 4.15 In Vitro Macrophage Polarization Assays

Raw 264.7 cells were seeded in 12‐well plates at a density of 1×10^5^ cells/well. Except for the Control group, all other groups were pretreated with LPS (1 µg/mL, 12 h) before receiving the following interventions: LPS group: LPS stimulation only, without further treatment; LPS+Bi_2_WO_6_:Yb,Er@CuS@CS+Dark group: After LPS stimulation, cells were treated with the Bi_2_WO_6_:Yb,Er@CuS@CS nanoplatform (200 µg/mL) and incubated in the dark; Bi_2_WO_6_:Yb,Er@CuS@CS+NIR group: Following LPS stimulation, cells were treated with the Bi_2_WO_6_:Yb,Er@CuS@CS nanoplatform (200 µg/mL) and subjected to NIR irradiation (980 nm, <1.0 W/cm^2^) for 15 min, maintaining a controlled temperature of 42 ± 0.5°C, followed by 24 h of incubation. The expression levels of M1‐related markers (TNF, iNOS, IL‐1β; listed in Table S4) and M2‐related markers (CD206, IL‐10, Arg‐1; listed in Table S4) were evaluated via RT‐qPCR. Additionally, cells were stained with APC‐conjugated anti‐CD86 (M1 marker) and PE‐conjugated anti‐CD206 (M2 marker) antibodies and analyzed using a Beckman CytoFLEX flow cytometry system. To further assess the immunomodulatory effects of mild hyperthermia, Raw 264.7 cells from the different treatment groups were fixed and stained with immunofluorescence dyes targeting iNOS (a marker of M1 macrophages) and Arg‐1 (a marker of M2 macrophages). Imaging was performed using a Leica DMI‐8 fluorescence microscope.

### 4.16 RNA Isolation and Quantitative Real‐Time Polymerase Chain Reaction (qRT‐PCR)

Total RNA was extracted from various cell lines using the RNA Purification Kit (EZBioscience), following the manufacturer's guidelines. cDNA synthesis was carried out with the Color Reverse Transcription Kit (EZBioscience). Quantitative real‐time PCR was subsequently performed on a QuantStudio 7 Flex System (Life Technologies) using the obtained cDNA, 2× Color SYBR Green qPCR Master Mix (EZBioscience), and primers supplied by BioTNT, in accordance with the recommended protocol. Gene expression levels were quantified via the 2^–ΔΔCt^ method. The corresponding primer sequences are listed in Tables S4 and S5.

### 4.17 Assessment of Macrophage Polarization In Vitro via Flow Cytometry

Raw 264.7 cells were plated in a 12‐well plate at a density of 2×10^5^ cells per well and treated under various conditions for 24 h. Subsequently, the cells were collected and prepared for flow cytometry. To distinguish between M1 and M2 macrophage phenotypes, staining was performed using APC‐conjugated anti‐mouse CD86 antibodies (BioLegend, USA) and PE‐conjugated anti‐mouse CD206 antibodies (BioLegend, USA). Data acquisition was performed using a flow cytometer (Beckman Coulter, Brea, CA, USA).

### 4.18 In Vitro Immunofluorescence Staining of Macrophages

Raw 264.7 cells were plated in confocal dishes at a density of 1×10^5^ cells per dish and subjected to various treatments for 24 h. Following culture, the cells were fixed with 4% paraformaldehyde for 15 min, washed three times with PBS, and then permeabilized using 0.1% Triton X‐100 for 15 min. After blocking for 1 h, the samples were incubated with primary antibodies against iNOS or Arg‐1 (Abcam, UK), followed by corresponding secondary antibodies. Imaging was performed using a confocal microscope.

### 4.19 In Vitro Angiogenesis Assay

To evaluate the potential proangiogenic effects of the Bi_2_WO_6_:Yb,Er@CuS@CS nanoplatform (200 µg/mL), we employed a dual experimental approach: (1) direct coculture with HUVECs under various treatment conditions, and (2) culture of HUVECs with CM derived from differentially treated macrophages. HUVECs were seeded in 6‐well plates at a density of 2×10^5^ cells/well and incubated for 24 h before the following treatments: G1: DMEM complete medium only (control); G2: Coculture with Bi_2_WO_6_:Yb,Er@CuS@CS under dark conditions; G3: Coculture with Bi_2_WO_6_:Yb,Er@CuS@CS combined with mild photothermal therapy (PTT); G4: Macrophage‐derived CM without treatment; G5: CM from LPS‐stimulated macrophages; G6: CM from LPS‐stimulated macrophages cocultured with Bi_2_WO_6_:Yb,Er@CuS@CS under dark conditions; G7: CM from LPS‐stimulated macrophages cocultured with Bi_2_WO_6_:Yb,Er@CuS@CS and subjected to MTT. Following 24 h of treatment, an in vitro scratch assay was performed to assess cell migration. Wound closure was monitored at 0 h and 24 h postscratch, with migration rates calculated accordingly. For Transwell migration assays, HUVECs (1×10^4^ cells) were seeded in the upper chambers (8 µm pore size) with 500 µL of the respective treatment media in the lower chambers. After 24 h of incubation, nonmigrated cells were removed from the upper membrane surface, and the migrated cells were fixed, stained with crystal violet, and quantified.

To evaluate tube formation capacity, Matrigel assays were conducted by coating 24‐well plates with 100 µL/well of Matrigel matrix. Following polymerization at 37°C, HUVECs (1×10^5^ cells/well) from each treatment group (G1–G7) were seeded and incubated for 8 h. Tube formation was visualized by phase‐contrast microscopy, with quantitative analysis of tube length and branching nodes performed using ImageJ software.

VEGF expression (listed in Table S5) was assessed via RT‐qPCR and immunofluorescence staining as previously described. These comprehensive in vitro angiogenesis assays provide quantitative evaluation of the nanoplatform's effects on endothelial cell migration, tube formation, and proangiogenic factor expression.

### 4.20 Establishment of MRSA‐Infected Cutaneous Wound Model

All animal experiments were approved by the Institutional Animal Care and Use Committee of Shanghai Sixth People's Hospital Affiliated to Shanghai Jiao Tong University School of Medicine (Approval No: DWLL2025‐0015). Six‐week‐old male C57BL/6 mice were randomly selected and anesthetized via intraperitoneal injection of 1% sodium pentobarbital. A full‐thickness excisional wound (8 mm diameter) was created on the dorsal skin, followed by inoculation with 10 µL of Methicillin‐resistant *Staphylococcus aureus* (MRSA) suspension (1×10^6^ CFU/mL). The wound was then covered with Tegaderm film (3M, USA) to establish an infected cutaneous defect model.

The treatment regimen consisted of two phases: Anti‐infection phase (Day 0–Day 4): A single antibacterial treatment was administered, involving topical application of Bi_2_WO_6_:Yb,Er@CuS@CS nanoplatform followed by 30 min of SSE and 15 min of 980 nm, 1 W/cm^2^ NIR irradiation; Wound healing phase (Day 4–Day 14): Mild thermal therapy (MTT, 42 ± 0.5°C for 15 min) was performed every 3 days under infrared thermographic guidance, with the nanoplatform reapplied before treatment. All mice were randomly divided into 5 groups (G1–G5) for differential intervention. The specific grouping was as follows: G1(Control): PBS only (no nanoplatform or irradiation); G2 (Nanoplatform only): Bi_2_WO_6_:Yb,Er@CuS@CS without SSE/NIR or MTT; G3 (Anti‐infection only): Full antibacterial treatment (Day 2) + PBS during healing phase; G4 (Nanoplatform without MTT): Antibacterial treatment + nanoplatform in healing phase (no MTT); G5 (combined therapy): Antibacterial treatment + nanoplatform + MTT during healing phase. Wound closure and infection status were monitored on Days 2, 4, 8, and 14, with photographic documentation and quantification of wound healing rates.

### 4.21 Bacterial Load Assessment

On Day 4, infected wound tissues (1 g) were homogenized in PBS, serially diluted (100×), and plated on SBA. After 24 h incubation at 37°C, MRSA colony‐forming units (CFUs) were enumerated.

### 4.22 Histopathological and Immunohistochemical Analysis

Tissues were harvested on Day 7 and Day 14 postwounding. Animals were euthanized via intraperitoneal injection of an overdose of sodium pentobarbital, and infected wound tissues along with adjacent normal skin were harvested for histological analysis. The collected specimens were fixed in 4% paraformaldehyde for 24 h, embedded in paraffin, and sectioned for subsequent staining procedures. The following staining protocols were performed: H&E staining for general histopathological evaluation; Masson's trichrome staining for analysis of collagen deposition and Giemsa staining for detection of residual bacteria. Immunofluorescence staining was conducted to assess: Macrophage polarization using iNOS (M1 marker) and Arg‐1 (M2 marker) antibodies; Angiogenic potential using CD31 (endothelial cell marker) and α‐SMA (pericyte marker) antibodies. Immunohistochemical staining was performed to evaluate VEGF expression levels as an indicator of angiogenic activity and TNF‐α expression as a marker of inflammatory response. Additionally, major organs (heart, liver, spleen, lung, and kidney) were randomly collected from each experimental group for H&E staining to assess the in vivo biosafety profile of the Bi_2_WO_6_:Yb,Er@CuS@CS nanoplatform.

### 4.23 Statistical Analysis

All data were derived from ≥ 3 independent experiments. Statistical analyses were conducted using GraphPad Prism 8 software (version 8.0.2). Continuous variables are reported as mean ± standard deviation. One‐way ANOVA with Tukey's post hoc test was employed for between‐group comparisons, with statistical significance set at *P* < 0.05.

## Conflicts of Interest

The authors declare no conflicts of interest.

## Supporting information




**Supporting File 1**: advs74966‐sup‐0001‐SuppMat.docx.

## Data Availability

Research data are not shared.
