# Supplementary material for: Synergistic Sunlight‐Activated Photodynamic and Near‐Infrared‐Induced Mild Photothermal Therapy for Infected Wound Healing Using Functionalized Nano‐Bi2WO6 Composites
Source: Adv Sci (Weinh). 2026 Apr 10;13(32):e22124. doi: 10.1002/advs.202522124 (PMC13252625; doi:10.1002/advs.202522124)
Supplement: Supplementary file 1 — Supporting File 1: advs74966‐sup‐0001‐SuppMat.docx. [file ADVS-13-e22124-s001.docx]

Supporting Information

**Synergistic Sunlight-Activated Photodynamic and Near-Infrared-Induced Mild Photothermal Therapy for Infected Wound Healing Using Functionalized Nano-Bi_2_WO_6_ Composites**

*Yihao Sun, Liangrui He, Zhijun Shen, Yuxin Zhang, Yizhang Tang, Xujiang Yu, Jisi Zheng***, Moran Huang***, Wanwan Li***, Lei Wang**

1. Supplementary Figures


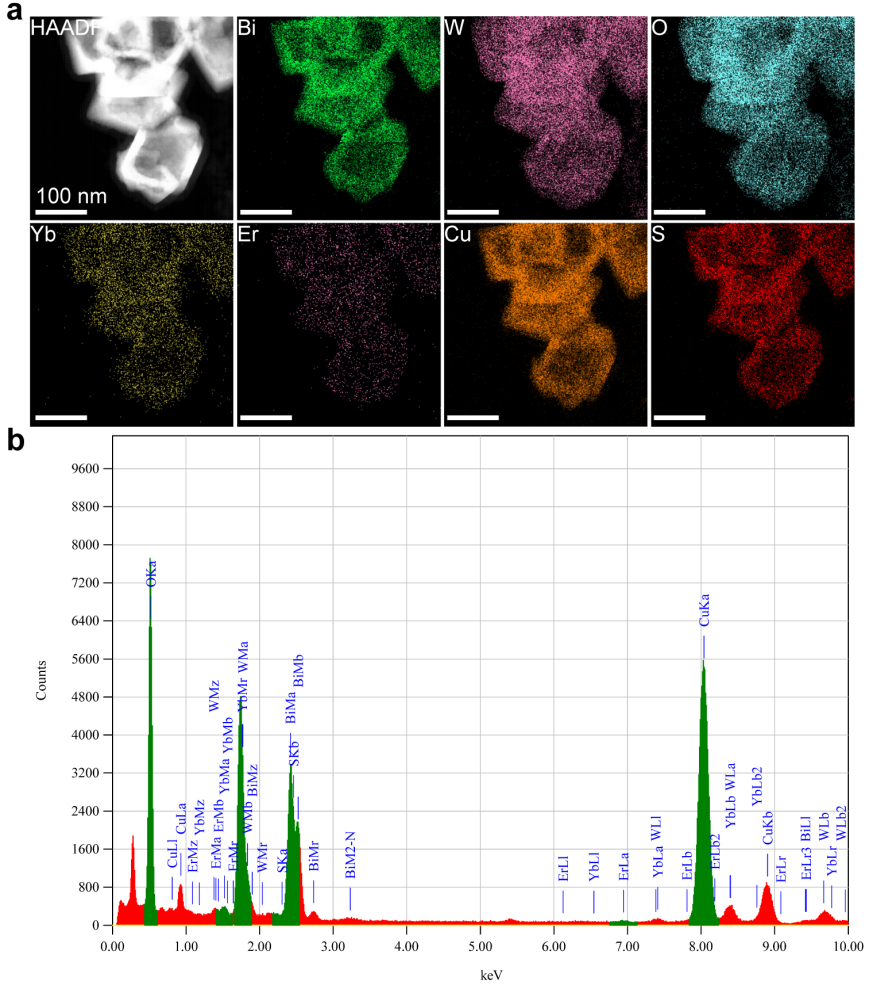


Figure S1. The EDS mapping and line scan of BWO:Yb,Er@SiO_2_@CuS


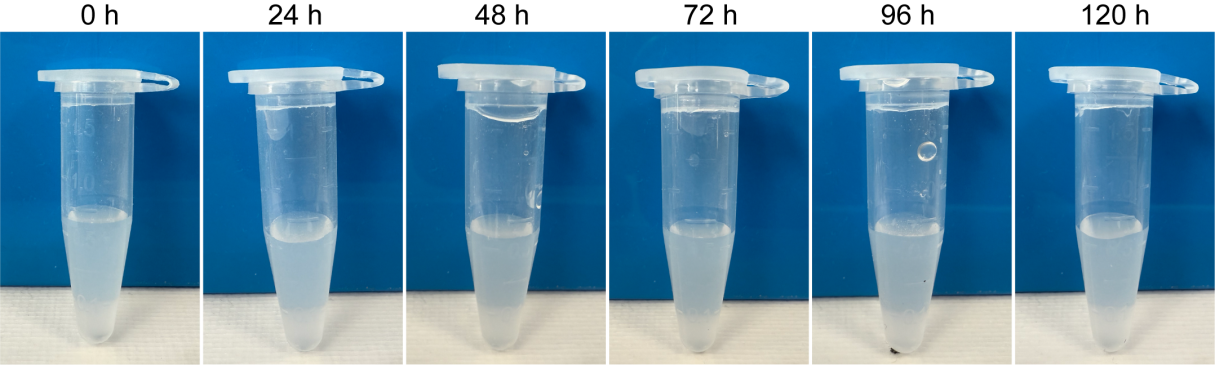


**Figure S2.** Stability of Bi_2_WO_6_:10%Yb,2%Er@CuS@CS nanoplatforms (in MES buffer, pH=4.7) at different time.


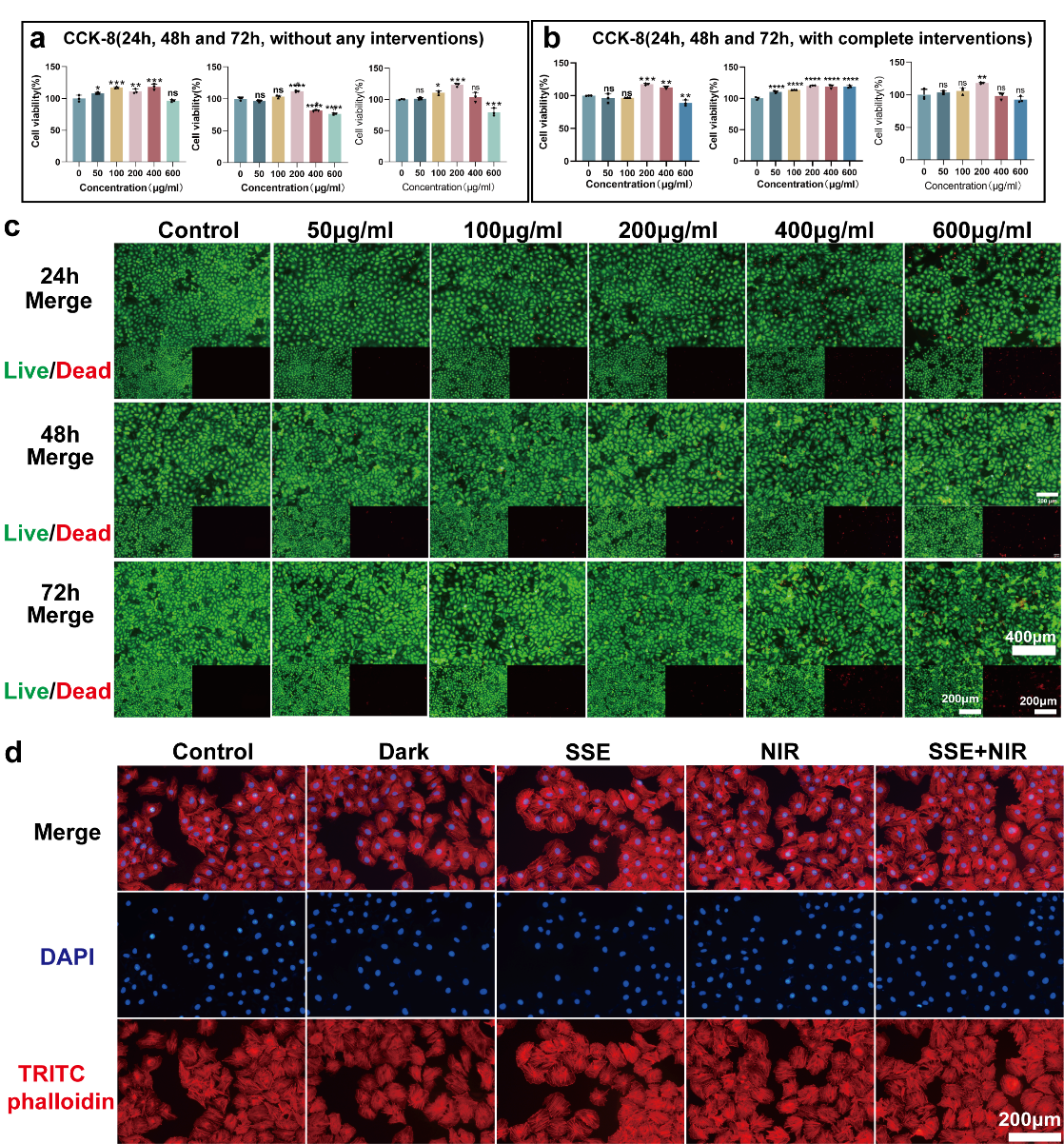


**Figure S3.** Bi_2_WO_6_:Yb,Er@CuS@CS nanoplatform exhibits excellent biocompatibility. (a) Cell viability of HUVECs co-cultured with the nanoplatform alone at 24h (left), 48h (middle), and 72h (right), n=3; (b) Cell viability of HUVECs co-cultured with the nanoplatform and subjected to complete intervention at 24h (left), 48h (middle), and 72h (right), n=3. The full intervention consisted of 30 minutes of Simulated Sunlight Exposure (SSE) and 15 minutes of 1.0 W/cm^2^ Near-Infrared Radiation (NIR); (c) Representative live/dead staining images of HUVECs co-cultured with different concentrations of the nanoplatform and subjected to full intervention, including 24h, 48h and 72h post-intervention. Live cells: green; dead cells: red; (d) TRITC phalloidin staining of actin cytoskeleton structure in HUVECs treated with 200 μg/ml nanoplatform for 24h after different interventions. TRITC phalloidin (red): actin cytoskeleton; DAPI (blue): nuclei. (ns, p>0.05; *, p<0.05; **, p<0.01; ***, p<0.001; ****, p<0.0001)


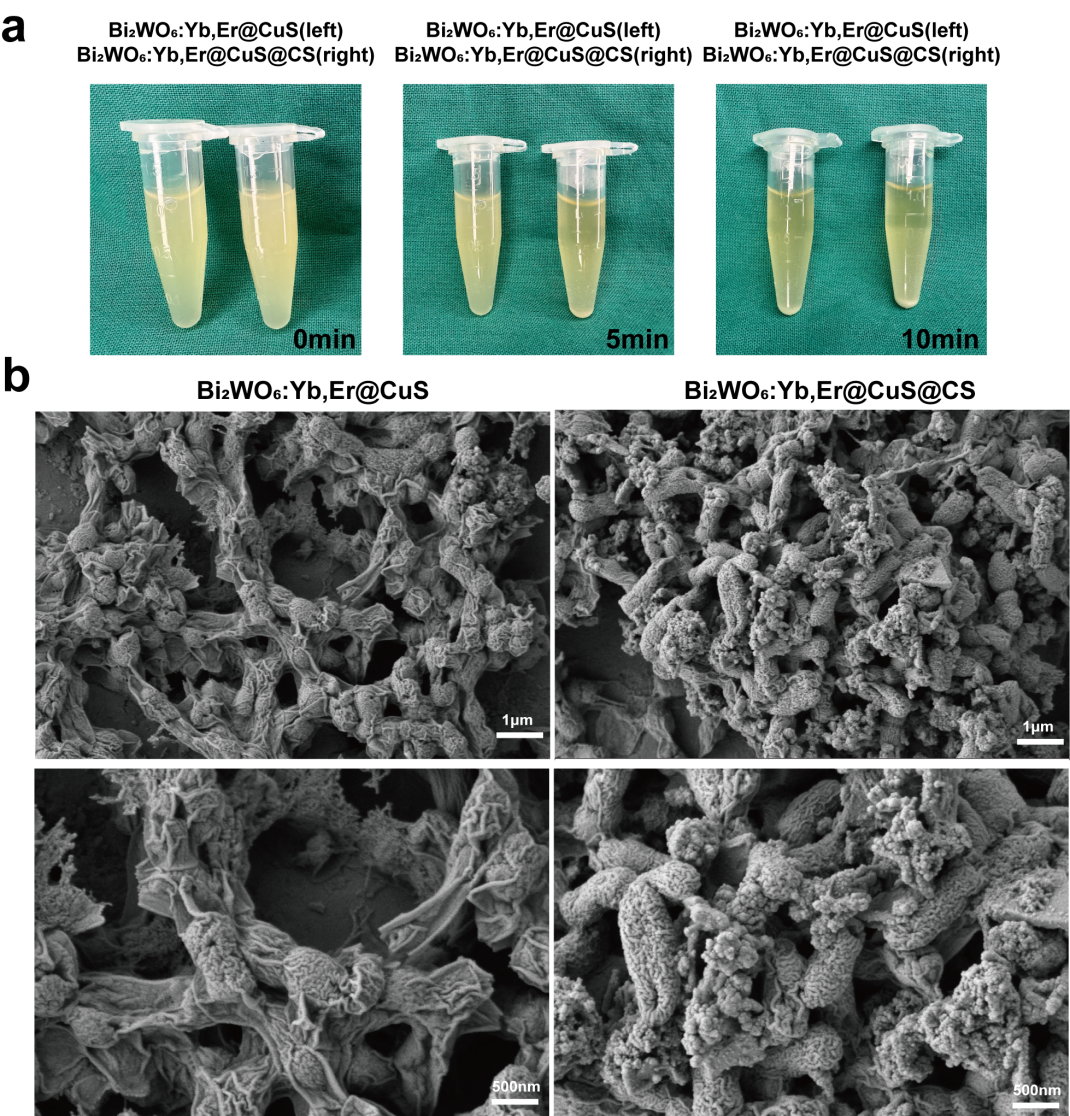


**Figure S4.** (a) Mixed bacterial suspensions of MRSA and E. coli (10^8^ CFU/mL) were thoroughly mixed with Bi_2_WO_6_:Yb,Er@CuS@CS (200 μg/mL) and Bi_2_WO_6_:Yb,Er@CuS (200 μg/mL), respectively, and photographed after standing for 0 minutes, 5 minutes, and 10 minutes. (b) SEM images of mixed bacterial biofilms after different treatments.


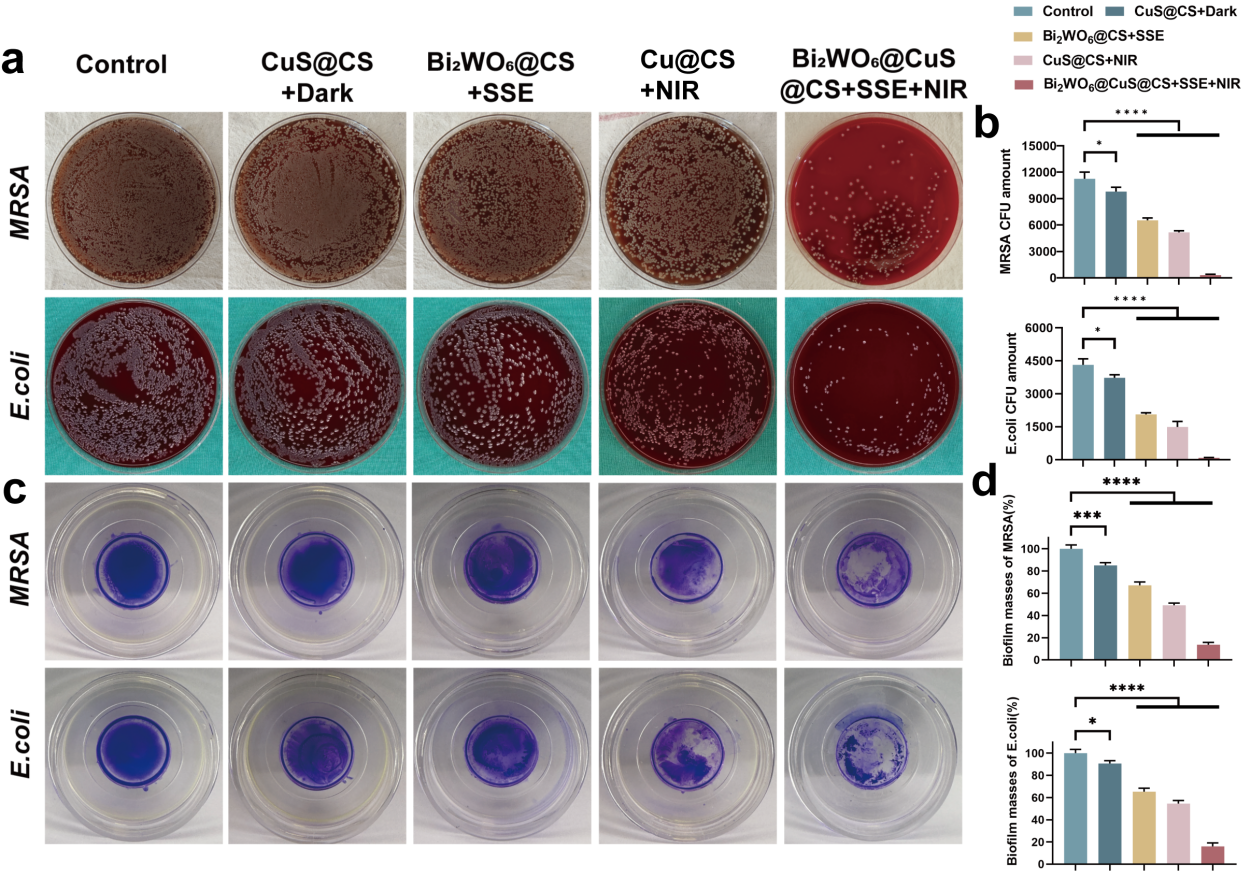


**Figure S5**. The antibacterial efficacy of the ROS from sunlight (PDT), heat from NIR light (PTT), and Cu^2+^ release. (a) Photographs of bacterial colonies grown on sheep blood agar plates from different treatment groups, with (b) quantitative analysis of CFU counts, n=3; (c) Crystal violet staining images of biofilms from different treatment groups, with (d) absorbance measurements of stained biofilms at 550 nm, n=3. (ns, p>0.05; *, p<0.05; **, p<0.01; ***, p<0.001; ****, p<0.0001)


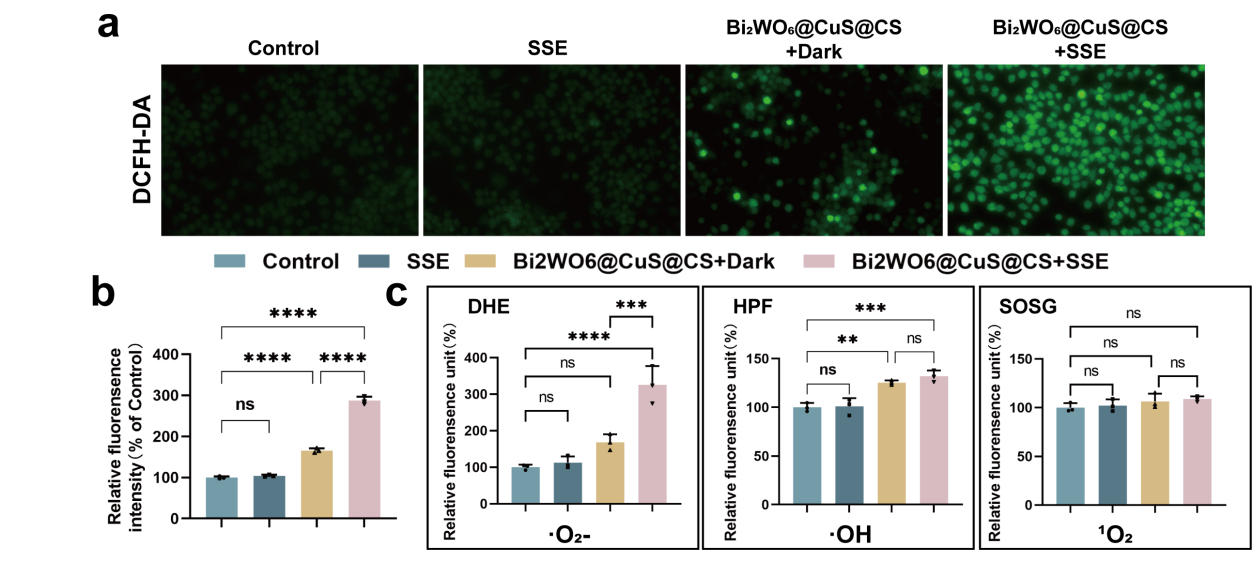


Figure S6. (a) Representative fluorescence images of ROS, with (b) quantitative analysis of ROS, n=3; (c) Classification of the generated ROS types: superoxide anion (·O_2_⁻), hydroxyl radical (·OH), and singlet oxygen (^1^O_2_) were distinguished using three fluorescent probes—DHE, HPF, and SOSG, respectively. n=3. (ns, p>0.05; *, p<0.05; **, p<0.01; ***, p<0.001; ****, p<0.0001)


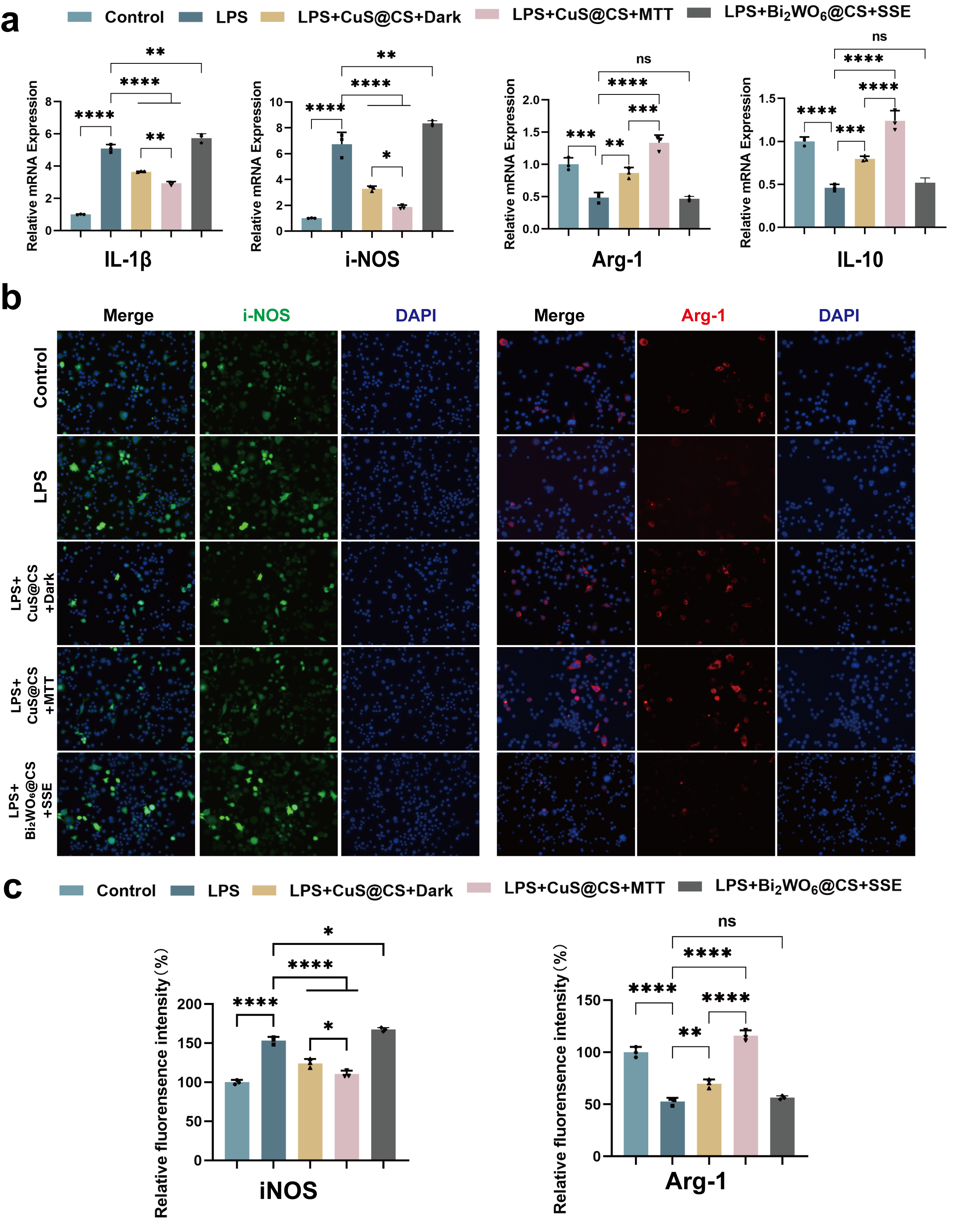


**Figure S7.** The immunomodulatory effects of the Cu^2+^ release, mild heat from NIR light (MTT), and ROS from sunlight (PDT) on macrophages. (a) mRNA expression levels of M1 macrophage markers (i-NOS and IL-1β) and M2 macrophage markers (IL-10 and Arg-1), n=3; (b) Representative confocal microscopy images of RAW 264.7 cells under various treatments: i-NOS (green, M1 phenotype), Arg-1 (red, M2 phenotype); (c) Quantitative analysis of the immunofluorescence images in (b). (ns, p>0.05; *, p<0.05; **, p<0.01; ***, p<0.001; ****, p<0.0001)


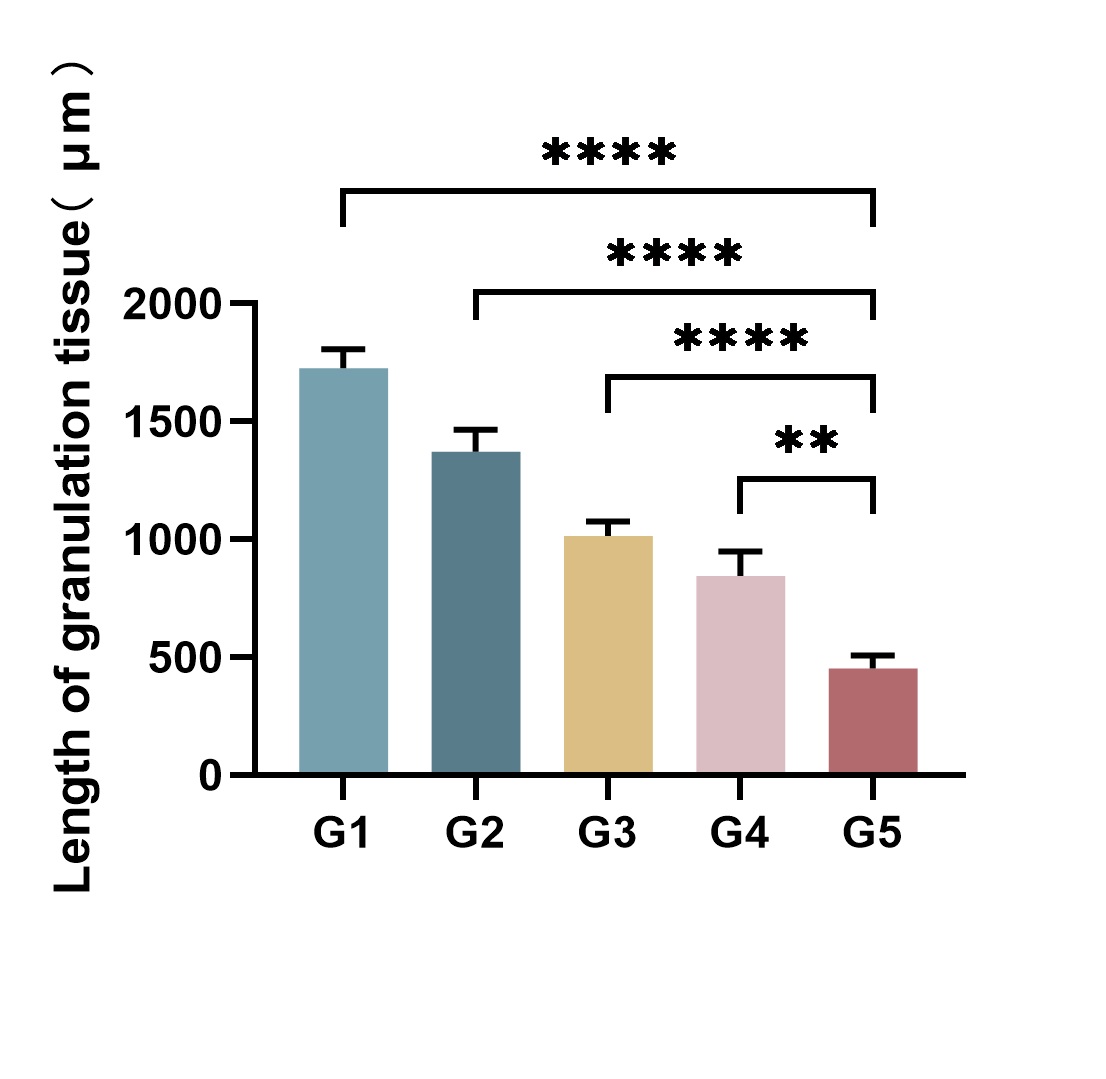


Figure S8 The length of granulation tissue on day 14. (**, p<0.01; ****, p<0.0001) (Grouping is consistent with the S2 annotation) (All mice were randomly divided into 5 groups (G1-G5) for differential intervention. The specific grouping was as follows: G1(Control): PBS only (no nanoplatform or irradiation); G2 (Nanoplatform only): Bi_2_WO_6_:Yb,Er@CuS@CS without SSE/NIR or MTT; G3 (Anti-infection only): Full antibacterial treatment (Day 2) + PBS during healing phase; G4 (Nanoplatform without MTT): Antibacterial treatment + nanoplatform in healing phase (no MTT); G5 (Combined therapy): Antibacterial treatment + nanoplatform + MTT during healing phase.)


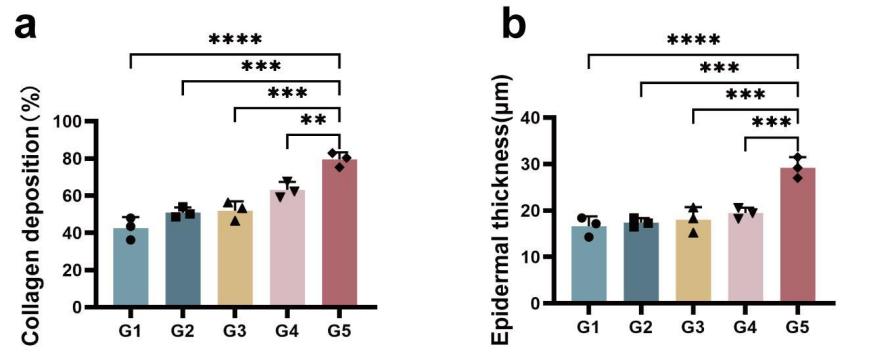


Figure S9. (a) Quantification of the areas of stained collagen fibers. (b) Quantification of the average epidermal thickness. (**, p<0.01; ***, p<0.001; ****, p<0.0001) (Grouping is consistent with the Figure S8 annotation)

**
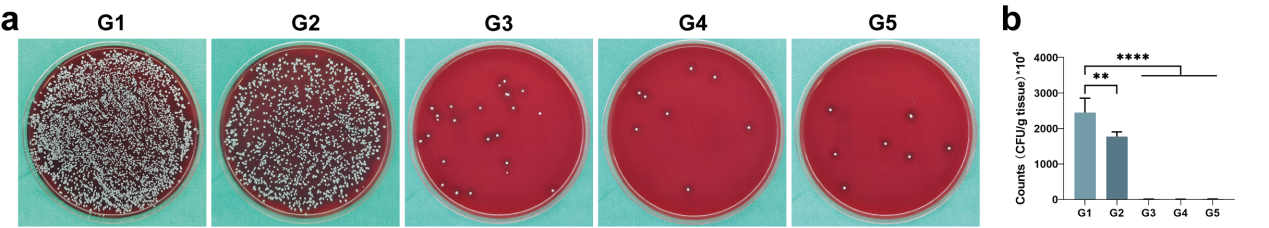
**

Figure S10. (a) Digital photos of bacterial colonies grown on sheep blood agar plates of wound tissues after homogenization. (b) Quantification of the numbers of CFU in (a). (**, p<0.01; ****, p<0.0001) (Grouping is consistent with the Figure S8 annotation)


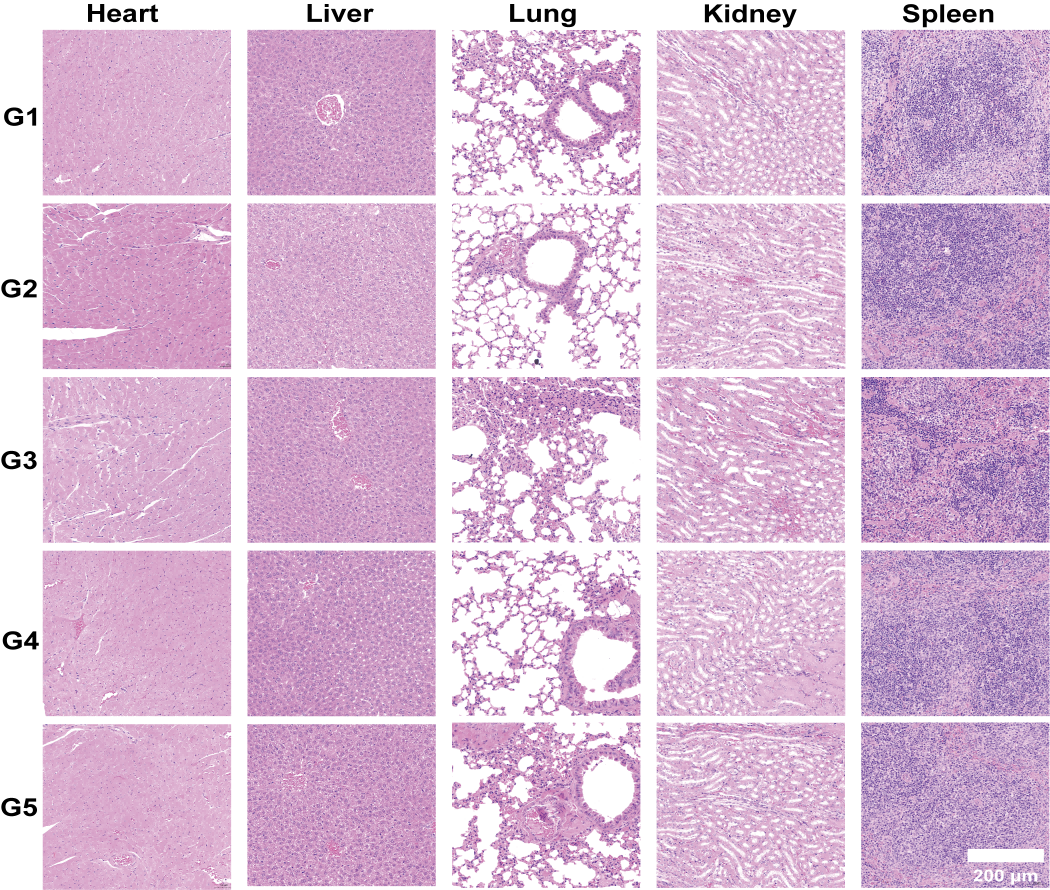


Figure S11. Biosafety evaluations of Bi_2_WO_6_:Yb,Er@CuS@CS intelligent nanoplatform in vivo. Representative H&E staining photos of organs such as heart, liver, lung, kidney, and spleen sections from mice of each group. (Grouping is consistent with the Figure S8 annotation)

1. **Supplementary Tables**

**Table S1**. ICP data of Bi_2_WO_6_:10%Yb,2%Er nanoparticles

|  | **Mass percent (%)** | **Atomic mass** | **Mole (*n*)** | **Molar fraction (%)** |
| --- | --- | --- | --- | --- |
| **Bi** | 41.58 | 208.98 | 0.00199 | 86.85 |
| **Yb** | 4.34 | 173.05 | 5.2*10^-5^ | 10.88 |
| **Er** | 0.87 | 167.26 | 0.00025 | 2.27 |

**Table S2**. ICP results for the supernatants of Bi_2_WO_6_:10%Yb,2%Er@CuS@CS nanoplatforms (in MES buffer, pH=4.7) centrifuged at different time points.

|  | **Bi (ppm)** | **W (ppm)** | **Yb (ppm)** | **Er (ppm)** | **Cu (ppm)** |
| --- | --- | --- | --- | --- | --- |
| **0 h** | 0.02 | 0.010 | 0.001 | 0.004 | 0.002 |
| **24 h** | 0.03 | 0.011 | 0.002 | 0.005 | 0.003 |
| **48 h** | 0.04 | 0.011 | 0.001 | 0.004 | 0.002 |
| **72 h** | 0.05 | 0.013 | 0.001 | 0.006 | 0.001 |
| **96 h** | 0.04 | 0.012 | 0.001 | 0.004 | 0.002 |
| **120 h** | 0.04 | 0.014 | 0.001 | 0.004 | 0.002 |

**Table S3.** Line scan results of Bi_2_WO_6_:10%Yb,2%Er

|  | **keV** | **Atom (%)** |
| --- | --- | --- |
| **Bi M** | 2.419 | 84.4 |
| **Yb M** | 1.521 | 14.5 |
| **Er L** | 6.947 | 1.1 |

**Table S4.** Primers used for qPCR of inflammatory genes

| **Gene** | **Upper primer sequence (5’ to 3’)** | **Lower primer sequence (5’ to 3’)** |
| --- | --- | --- |
| GAPDH | AAATGGTGAAGGTCGGTGTG | AGGTCAATGAAGGGGTCGTT |
| TNF-α | TAGCCAGGAGGGAGAACAGA | CCAGTGAGTGAAAGGGACAGA |
| iNOS | TTGACGCTCGGAACTGTA | GTTGGTGGCATAAAGTATGTG |
| IL-1β | TGGACCTTCCAGGATGAGGACA | GTTCATCTCGGAGCCTGTAGTG |
| Arg-1 | TGCTCACACTGACATCAACAC | GAGAATCCTGGTACATCTGGG |
| IL-10 | GCTCTTACTGACTGGCATGA | CGCAGCTCTAGGAGCATGTG |
| Mrc1 | CTCTGTTCAGCTATTGGACGC | TGGCACTCCCAAACATAATTTGA |

The primers were synthesized and provided by BioTNT.

**Table S5.** Primers used for qPCR of Angiogenesis-related genes

| **Gene** | **Upper primer sequence (5’ to 3’)** | **Lower primer sequence (5’ to 3’)** |
| --- | --- | --- |
| GAPDH | GGTCTCCTCTGACTTCAACAGCG | CCAAATTCGTTGTCATACCAGGAA |
| vegf | TACCTCACCAAAGCCAGCAC | CAGGACGGGCAGAATCATCA |

The primers were synthesized and provided by BioTNT.
